# Associations of dietary glycemic index and load during pregnancy with blood pressure, placental hemodynamic parameters and the risk of gestational hypertensive disorders

**DOI:** 10.1007/s00394-021-02670-5

**Published:** 2021-09-15

**Authors:** Clarissa J. Wiertsema, Rama J. Wahab, Annemarie G. M. G. J. Mulders, Romy Gaillard

**Affiliations:** 1 The Generation R Study Group, Erasmus MC, University Medical Center, PO Box 2040, 3000 CA Rotterdam, The Netherlands; 2 Department of Pediatrics, Sophia’s Children’s Hospital, Erasmus MC, University Medical Center, Rotterdam, The Netherlands; 3 Departments of Obstetrics and Gynecology, Erasmus MC, University Medical Center, Rotterdam, The Netherlands

**Keywords:** Glycemic index, Glycemic load, Pregnancy, Blood pressure, Gestational hypertensive disorders

## Abstract

**Purpose:**

The aim of this study was to examine the associations of dietary glycemic index and load with gestational blood pressure, placental hemodynamic parameters and the risk of gestational hypertensive disorders.

**Methods:**

In a population-based cohort among 3378 pregnant Dutch women, dietary glycemic index and load were assessed from food frequency questionnaires at median 13.4 (95% range 9.9–22.9) weeks gestation. Blood pressure was measured in early-, mid- and late-pregnancy. Placental hemodynamic parameters were measured in mid- and late-pregnancy by ultrasound. Data on gestational hypertensive disorders was acquired from medical records.

**Results:**

Mean dietary glycemic index (SD) was 58 (3) and mean dietary glycemic load (SD) was 155 (47). Dietary glycemic index was not associated with blood pressure, placental hemodynamic parameters and the risk of gestational hypertensive disorders. Higher dietary glycemic load SDS was associated with a higher diastolic blood pressure in early-pregnancy, remaining after adjustment for socio-demographic and lifestyle factors ((0.98 (95% CI 0.35–1.61) mmHg per SDS increase in glycemic load). No other associations of glycemic load with blood pressure or placental hemodynamic parameters and the risk of gestational hypertensive disorders were present. No significant associations of dietary glycemic index and load quartiles with longitudinal blood pressure patterns from early to late-pregnancy were present.

**Conclusion:**

Within this low-risk pregnant population, we did not find consistent associations of dietary glycemic index and load with blood pressure, placental hemodynamic parameters and the risk of gestational hypertensive disorders. Further studies need to assess whether the effects on gestational hemodynamic adaptations are more pronounced among high-risk women with an impaired glucose metabolism.

**Supplementary Information:**

The online version contains supplementary material available at 10.1007/s00394-021-02670-5.

## Introduction

Gestational hypertensive disorders affect up to 10% of pregnancies and are a major risk factor for maternal and neonatal morbidity and mortality [1]. Women with a medical history of gestational hypertensive disorders are at increased risk of chronic hypertension and cardiovascular disease in later life [2]. In non-pregnant populations, the quality and quantity of carbohydrate intake seem to influence blood pressure and other cardiovascular risk factors, including body weight, impaired lipid metabolism and insulin resistance [3–5]. The glycemic index and load are commonly used dietary measures to qualify carbohydrate intake, and provide information on the postprandial glycemic response to carbohydrate containing food products [6, 7]. A low-glycemic index diet can be achieved by consuming carbohydrate containing food products that are less likely to increase blood sugar levels referred to as low-glycemic index products, while avoiding products with a high-glycemic index. For a low-glycemic load diet the daily quantity of carbohydrates is additionally taken into account. A meta-analysis consisting of 14 intervention studies comprising 1097 healthy non-pregnant individuals with a mean age ranging from 28 to 54 years, showed that a daily glycemic index reduction of 10 units lowered systolic and diastolic blood pressure by 1.1 and 1.3 mmHg, respectively [3]. This meta-analysis also showed that a daily glycemic load reduction of 28 units lowered systolic and diastolic blood pressure by 2.0 mmHg [3].

During pregnancy, replacing high-glycemic index products by lower glycemic index products may also have favorable effects on pregnancy outcomes, especially among women at increased risk of an impaired glucose metabolism [8]. A low-glycemic index diet during pregnancy is suggested to have beneficial effects on glucose metabolism, lipid profile, gestational weight gain and the risk of delivering a large-for-gestational-age-infant [8–16]. Dietary glycemic index and load have a direct effect on postprandial glucose levels. Higher glucose levels during pregnancy can impair endothelial function through oxidative stress and vascular inflammation, with elevated blood pressure and impaired placental vascular function as a possible result predisposing to an increased risk of gestational hypertensive disorders [17–19]. Already it has been shown that higher glucose levels are associated with a higher risk of gestational hypertensive disorders [20]. However, not much is known about the effects of low-glycemic index and load diets on gestational hemodynamic adaptations and the risk of gestational hypertensive disorders. A case–control study in Iran among 202 pregnant women, showed that a daily dietary glycemic load above the median was associated with an increased risk of gestational hypertension [21]. Likewise, an intervention study in Italy among 370 overweight pregnant women found a lower incidence of gestational hypertension among women who were prescribed a low-glycemic index diet [22]. No previous studies have examined the influence of low-glycemic index and load diets on gestational blood pressure and placental hemodynamic adaptations, which are major determinants for the development of gestational hypertensive disorders.

We hypothesized that a lower dietary glycemic index and load during pregnancy positively influence hemodynamic adaptations during pregnancy, leading to a lower risk of gestational hypertensive disorders. Therefore, we examined the associations of dietary glycemic index and load with blood pressure and placental vascular function throughout pregnancy and the risks of gestational hypertensive disorders within a population-based cohort study among 3378 pregnant women.

## Methods

### Study design and study sample

**Fig. 1:**
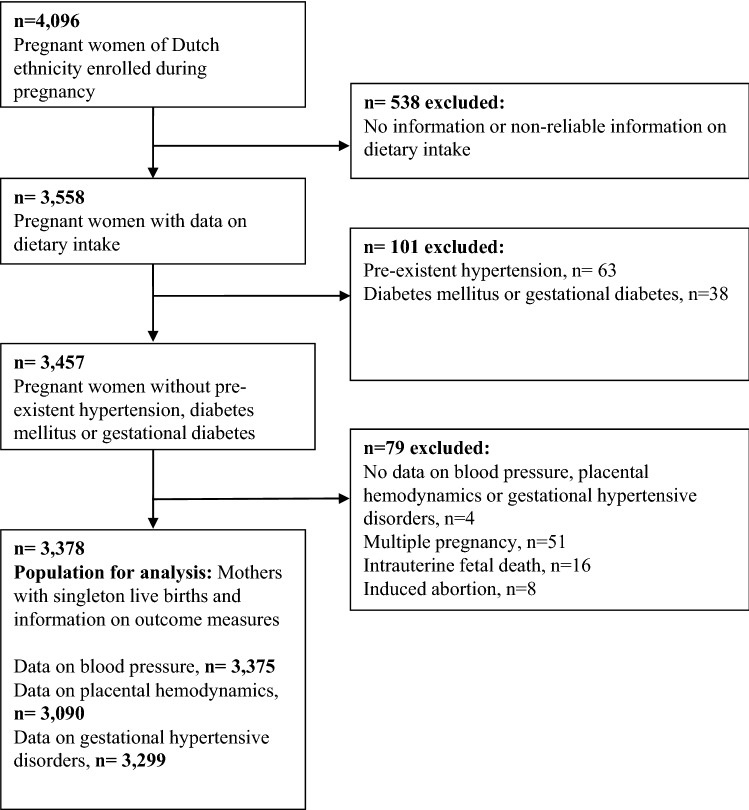
Flow chart of the study population

The study was embedded in the Generation R study, a population-based prospective cohort from early-pregnancy onwards in Rotterdam, The Netherlands [23, 24]. In total, 4096 Dutch women were enrolled during pregnancy. Information on dietary intake was available for 3558 women. We excluded women with pre-existent hypertension and diabetes, with missing outcome data, and non-singleton live-births (*n* = 180). The population for analysis consisted of 3378 pregnant women (Fig. 1). This study was performed in accordance with the ethical standard laid down in the Declaration of Helsinki and was approved by the Medical Ethical Committee of the Erasmus Medical Centre in Rotterdam, The Netherlands (MEC 198.782/2001/31). All participating women gave written informed consent prior to their inclusion in the study.

### Maternal dietary glycemic index and glycemic load

Semi-quantitative food frequency questionnaires (FFQ) consisting of 293 food items were obtained at study enrollment (median = 13.4 weeks of gestation, 95% range 9.9–22.9). The FFQ considered dietary intake of the three months prior and was validated in a subgroup of 83 Dutch women against three non-consecutive 24 h dietary recalls, with further confirmation using nutritional biomarkers [25]. Intraclass correlation coefficients between nutrient intake estimates from the FFQ and from the 24 h dietary recalls ranged from 0.47 to 0.77 for macronutrients, and was 0.60 for total carbohydrate intake. We calculated mean dietary glycemic index and load per day as described previously [26]. We used the dietary glycemic index as primary exposure, as this is most commonly used in clinical and research settings. As the dietary glycemic load additionally takes into account the daily quantity of carbohydrates consumed, it provides additional information on postprandial glucose levels but might also be more sensitive for measurement error [6, 7, 26].Glycemic index values were obtained from the glycemic index database on the Dutch diet by the Medical Research Council Human Nutrition Research, and assigned to each individual food item in the FFQ [27]. This database was developed using a standardized approach of calculating dietary glycemic index and load to facilitate research into the health effect of dietary glycemic index and load [27]. This approach is used in comparable observational studies that investigated the associations of dietary glycemic index and load with adverse birth and child outcomes [28, 29]. Mean dietary glycemic index per day was calculated by summing the product of the carbohydrate intake of each food item with its glycemic index, which was then divided by the total amount of carbohydrates consumed per day. The mean dietary glycemic load per day was calculated by summing the product of the carbohydrate intake of each food item with it glycemic index [6, 7, 26]. To examine whether associations were restricted to women with a relatively high dietary glycemic index or load within our study population, and to explore whether a linear tendency was present, we constructed quartiles of dietary glycemic index and load for our study population. Since a linear tendency was present, we also constructed standard deviation scores (SDS) of dietary glycemic index and load to assess the continuous associations of dietary glycemic index and load per 1-SDS increase with gestational hemodynamic developmental outcomes. Finally, to increase clinical interpretability, dietary glycemic index per day was categorized into categories using similar cut-offs as used for individual food product: low-glycemic index diet (≤ 55), a normal-glycemic index diet (56–69) and a high-glycemic index diet (≥ 70) as a secondary analysis [16, 26]. We consider this method in line with studies that recommend a low-glycemic index diet by replacing high-glycemic index food products with low-glycemic index food products as dietary intervention [26].

### Blood pressure in pregnancy

Systolic blood pressure and diastolic blood pressure were measured in early-, mid- and late-pregnancy (medians, 95% range 12.9 (9.8–17.2), 20.4 (18.6–23.2), 30.2 (28.6–32.6) weeks gestation, respectively) using an Omron 907 automated digital oscillometric sphygmomanometer (OMRON Healthcare Europe BV, Hoofddorp, The Netherlands) [30]. The participant was seated in upright position with feet on the floor. The cuff was placed around the non-dominant arm supported at the level of the heart. Blood pressure measurement started after a minimum of 5 min at rest. The mean systolic and diastolic blood pressure was calculated of two readings with a 60 s interval [31].

### Placental hemodynamic parameters

Placental hemodynamic parameters were measured in mid- and late-pregnancy (medians, 95% range 20.5 (18.8–22.9), 30.4 (28.5–32.6) weeks gestation, respectively) using a detailed ultrasonography protocol [32, 33]. The umbilical artery pulsatility index (UmPI) was measured in a free-floating part of the umbilical cord and the uterine artery resistance index (UtRI) at the crossover with the external iliac artery. Three sequential flow velocity wave forms were recorded with the mean of three Doppler measurements being used for further analysis. Bilateral uterine artery notching was defined as an upturn of the velocity waveform at the beginning of diastole in both uterine arteries [33].

### Gestational hypertensive disorders

Information on preeclampsia and gestational hypertension was obtained from medical records and cross checked with the original hospital charts, as described previously [34, 35]. Gestational hypertension was defined as a systolic blood pressure of at least 140 mmHg and/or diastolic blood pressure of at least 90 mmHg after 20 weeks of gestation in previously normotensive women. These criteria including the manifestation of proteinuria were used to identify preeclampsia [36].

### Covariates

Data on maternal age, education level, parity, prepregnancy weight, folic acid supplement use, alcohol consumption during pregnancy, smoking during pregnancy, and the diagnosis of pre-existent hypertension, pre-existent diabetes mellitus and gestational diabetes mellitus were collected by questionnaires during pregnancy. Information on dietary factors were obtained from the FFQ. Height was measured at enrolment to calculate the prepregnancy body mass index (BMI).

### Statistical power

As previously described, statistical power was calculated based on 7000 subjects within the Generation R Study {Jaddoe, 2006 #110; For a normally distributed continuous outcome a difference of 0.08 SD is detectable with type I error of 5% and a type 2 error of 20% (power of 80%), if 25% of the study population is exposed. This corresponds with an approximate difference of 0.90 mmHg for systolic and 0.70 mmHg for diastolic blood pressure. For gestational hypertensive disorders an odds ratio of 1.26 is detectable if 25% is exposed.

### Statistical analyses

First, we performed a non-response analysis to compare women with information on dietary glycemic index and load to those without to explore whether differences in characteristics between those women are present. Second, we examined the associations of glycemic index and load quartiles with longitudinal blood pressure patterns throughout pregnancy using unbalanced repeated measurement models. These models take into account the correlation of measurements within the same participant and allow for incomplete outcome data {Goldstein, 1995}. We constructed the best fitting model using fractional polynomials [37]. These models can be written as follows: Systolic blood pressure: *ß*_0_ + *ß*_1_ × GI/GL quartile + *ß*_2_ × gestational age + *ß*_3_ × gestational age^−2^ + *ß*_4_ × GI/GL quartile × gestational age. Diastolic blood pressure: *ß*_0_ + *ß*_1_ × GI/GL quartile + *ß*_2_ × gestational age + *ß*_3_ × gestational age^0.5^ + *ß*_4_ × GI/GL quartile × gestational age. In these models, ‘*ß*_0_ + *ß*_1_ × GI/GL quartile’ reflects the intercept. The intercept reflects the mean systolic and diastolic blood pressure value for the glycemic index and load categories. ‘*ß*_2_ × gestational age + *ß*_3_ × gestational age^−2’^ reflects the slope of change in systolic blood pressure per week, and ‘*ß*_2_ × gestational age + *ß*_3_ × gestational age^0.5^’, reflects the slope of change in diastolic blood pressure per week. Our term of interest is ‘*ß*_4_ × GI/GL quartile × gestational age’, which reflects the difference in blood pressure change per week per glycemic index or load quartile, as compared to women in the lowest glycemic index or load quartile. As a second step, we examined the associations of dietary glycemic index and load SDS and quartiles with differences in early-, mid- and late-pregnancy blood pressure separately using linear regression models to identify potential critical periods in gestational hemodynamic adaptions important from an etiological perspective. Third, we examined the associations of dietary glycemic index and load in SDS and quartiles with differences in umbilical artery pulsatility index and uterine artery resistance index in mid- and late-pregnancy using linear regression models and the risk of bilateral uterine artery notching using logistic regression models. Finally, we examined the risk on gestational hypertensive disorders using logistic regression models.

Potential confounding by maternal socio-demographic and lifestyle factors needs to be taken into account as it is well-known that dietary intake is strongly related to these other maternal characteristics. Potential confounders were selected beforehand using a directed acyclic graph (Supplementary figure S1). We constructed four different adjustment models as it is well-known that dietary exposures are strongly related to socio-demographic, lifestyle and other dietary factors, which may explain potential associations. (1) Basic model, in which we adjusted for gestational age at intake; (2) Socio-demographic model, in which we additionally adjusted for maternal age, educational level and parity; (3) Lifestyle model in which we additionally adjusted for prepregnancy BMI, folic acid use, smoking habits and alcohol use, and total energy intake; (4) Dietary model: in which we additionally adjusted for dietary fiber intake, salt intake and gestational weight gain if we found significant associations in the lifestyle model. These dietary factors are closely linked to dietary glycemic index and load, and may also influence the development of gestational hypertensive disorders. Covariates were included in the models if they were associated with both outcome and exposure (*p* value < 0.05 and > 10% change in effect estimate when added to the univariate model) [38].

We conducted four sensitivity analyses: (1) We repeated the analyses for dietary glycemic index using a cut-off to classify diets into a low, normal or high-glycemic index diet; (2) We repeated the analyses restricted to women with a prepregnancy BMI ≥ 25, as they represent a population at higher risk of impaired glucose metabolism who may be more prone to adverse effects of a higher dietary glycemic index and load diet; (3) We repeated the analyses restricted to participants who were enrolled in early-pregnancy (i.e. < 14 weeks of gestation) as adherence to a lower dietary glycemic index and load already during preconception and early-pregnancy may have stronger effects on gestational hemodynamic adaptations. (4) We repeated the main analyses among participants with complete data on all covariates (non-imputed data). *P* values < 0.05 were considered as statistical significant. We used data from multiple imputations to reduce potential bias due to missing values of covariates. We used the Fully Conditional Specifications (FCS) method. In the imputation model all covariates and outcomes were included as predictor variables, and maternal weight and height at enrolment, paternal age and BMI, family income status, gestational age at birth and birth weight were included as additional predictor variables. We created five independent datasets, that were analyzed together and presented the pooled effect estimates. Analysis were performed using IBM Statistical Package of Social Sciences version 25. The analysis for repeated measurements was performed using Statistical Analysis System version 9.4.

## Results

### Participant characteristics

Table 1 shows that the mean dietary glycemic index (SD) was 57.7 (3.3) and the mean dietary glycemic load (SD) was 154.7 (46.9). A low-glycemic index diet according to the individual food product classification was consumed by 1059 (31%) pregnant women, whereas only one woman consumed a high-glycemic index diet according to the individual food product classification. No consistent differences were present in characteristics between women with information on dietary glycemic index and load to those without this information (Supplementary Table S1).

**Table 1:** Characteristics of the study population by glycemic index quartile (*n* = 3378)

	Total group	Glycemic index quartile 1	Glycemic index quartile 2	Glycemic index quartile 3	Glycemic index quartile 4	*p* value^c^
	*n* = 3378	*n* = 844	*n* = 845	*n* = 845	*n* = 844	
Maternal age at enrollment, years	31.4 (4.4)	32.4 (4.0)	31.6 (4.1)	31.1 (4.4)	30.4 (4.8)	< 0.001
Gestational age at intake, weeks	13.5 (5.4–38.1)	13.8 (10.5–23.2)	13.6 (10.0–24.4)	13.5 (9.8–23.1)	13.5 (9.8–22.6)	0.19
Parity, *n* nulliparous	2019 (59.9)	523 (62.1)	524 (62.0)	500 (59.3)	472 (56.2)	0.04
Prepregnancy BMI, kg/m^2^	23.1 (3.8)	22.8 (3.5)	23.0 (3.7)	23.2 (4.0)	23.3 (3.9)	0.04
Prepregnancy BMI ≥ 25, *n*	636 (21.8)	134 (18.2)	147 (20.3)	173 (23.7)	182 (25.1)	0.005
Gestational weigh gain, kg	10.8 (4.4)	10.6 (4.1)	10.8 (4.5)	10.9 (4.4)	10.7 (4.7)	0.59
Education, *n* high	1985 (59.5)	593 (70.8)	527 (62.9)	481 (58.0)	384 (46.3)	< 0.001
Glycemic index, per day	57.7 (3.3)	53.8 (1.4)	56.5 (0.6)	58.6 (0.7)	62.1 (1.9)	< 0.001
Glycemic load, per day	154.7 (46.9)	132.2 (32.8)	147.7 (38.7)	159.5 (43.5)	179.5 (55.9)	< 0.001
Carbohydrate intake, g/d	267.0 (75.1)	245.7 (60.4)	261.5 (68.5)	272.1 (74.1)	288.9 (88.3)	< 0.001
Protein intake, g/d	79.1 (19.1)	82.1 (18.1)	80.6 (18.9)	78.8 (19.1)	74.9 (19.6)	< 0.001
Fat intake, g/d	86.4 (24.3)	85.5 (23.7)	87.4 (24.0)	87.9 (24.6)	84.8 (24.8)	0.02
Fiber intake, g/d	23.4 (6.9)	25.1 (7.0)	24.1 (6.7)	23.1 (6.6)	21.4 (6.8)	< 0.001
Total energy intake, kcal/d	2146.3 (511.0)	2061.4 (451.8)	2137.6 (495.6)	2180.6 (516.1)	2205.4 (563.3)	< 0.001
Smoking, *n* continued during pregnancy	531 (17.0)	88 (11.3)	109 (13.9)	135 (17.2)	199 (25.4)	< 0.001
Alcohol, *n* continued during pregnancy	1559 (50.2)	442 (56.8)	404 (52.1)	369 (47.4)	344 (44.4)	< 0.001
Early-pregnancy, ≥ 1 glass per week	830 (26.9)	256 (30.8)	207 (24.9)	192 (23.1)	175 (21.1)	< 0.001
Mid-pregnancy, ≥ 1 glass per week	378 (12.3)	119 (31.5)	95 (25.1)	96 (25.4)	68 (18.0)	< 0.001
Late-pregnancy ≥ 1 glass per week	444 (14.7)	142 (32.0)	118 (26.6)	104 (23.4)	80 (18.0)	< 0.001
Folic acid supplement use, *n* yes	467 (89.1)	642 (90.9)	626 (89.7)	627 (91.3)	572 (84.5)	< 0.001
Systolic blood pressure, mmHg						
Early-pregnancy	117.1 (11.8)	116.3 (11.1)	116.9 (11.7)	118.0 (12.1)	117.4 (12.3)	0.05
Mid-pregnancy	118.4 (11.7)	117.0 (11.3)	118.5 (11.2)	119.1 (11.6)	118.9 (12.6)	0.001
Late-pregnancy	120.3 (11.4)	119.1 (10.7)	120.3 (11.2)	120.7 (11.4)	121.0 (12.1)	0.005
Diastolic blood pressure, mmHg						
Early-pregnancy	68.4 (9.2)	68.0 (8.9)	68.2 (8.9)	68.6 (9.7)	68.8 (9.0)	0.30
Mid-pregnancy	67.1 (9.2)	66.4 (8.7)	66.7 (8.6)	67.7 (9.9)	67.7 (9.7)	0.004
Late-pregnancy	69.3 (9.1)	68.7 (9.0)	69.4 (8.7)	69.3 (9.5)	69.7 (9.4)	0.181
Uterine artery resistance index						
Mid-pregnancy	0.535 (0.089)	0.540 (0.093)	0.533 (0.087)	0.533 (0.087)	0.534 (0.088)	0.49
Late-pregnancy	0.483 (0.078)	0.486 (0.082)	0.482 (0.075)	0.481 (0.073)	0.482 (0.080)	0.76
Umbilical artery pulsatility index						
Mid-pregnancy	1.188 (0.183)	1.178 (0.173)	1.194 (0.187)	1.198 (0.184)	1.182 (0.189)	0.16
Late-pregnancy	0.977 (0.166)	0.970 (0.171)	0.971 (0.159)	0.981 (0.161)	0.985 (0.174)	0.22
Late-pregnancy uterine artery notching	48 (2.2)	15 (2.7)	11 (2.0)	6 (1.1)	16 (3.0)	0.147
Gestational hypertensive disorders						
Gestational hypertension	166 (5.1)	38 (4.7)	45 (5.5)	39 (4.8)	44 (5.5)	0.84
Preeclampsia	58 (1.9)	16 (2.0)	11 (11.4)	18 (2.3)	13 (1.7)	0.57

Values are means (SD), median (95% range), or number (valid %)

^a^*p* values were obtained by chi-square tests for categorical variables, one-way ANOVA for continuous variables

### Dietary glycemic index and load with blood pressure throughout pregnancy

**Fig. 2:**
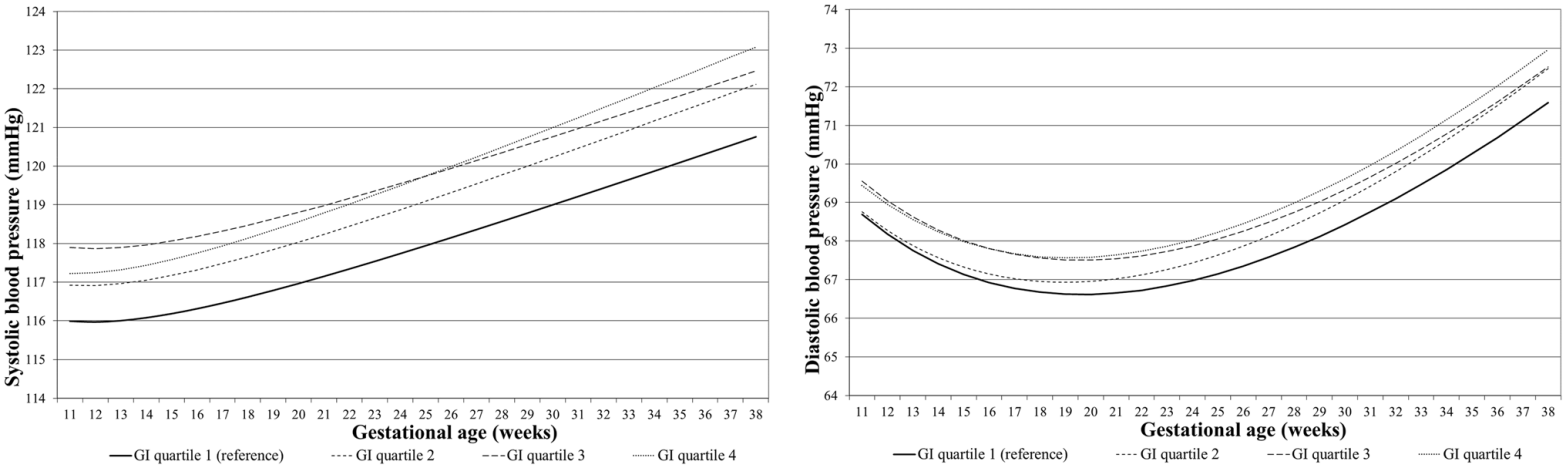
Blood pressure patterns in different glycemic index (GI) quartiles from repeated measurement models. Change in SBP and DBP in mmHg for first quartile, second quartile, third quartile and fourth quartile. SBP = *ß*_0_ + *ß*_1_ × GI quartile + *ß*_2_ × gestational age + *ß*_3_ × gestational age^−2^ + *ß*_4_ × GI quartile × gestational age. DBP = *ß*_0_ + *ß*_1_ × GI quartile + *ß*_2_ × gestational age + *ß*_3_ × gestational age^0.5^ + *ß*_4_ × GI quartile × gestational age. In these models, ‘*ß*_0_ + *ß*_1_ × GI’ reflects the intercept and ‘*ß*_2_ × gestational age + *ß*_3_ × gestational age^−2^ ‘reflects the slope of change in blood pressure per week for SBP, and ‘*ß*_2_ × gestational age + *ß*_3_ × gestational age^0.5^’, reflects the slope of change in blood pressure per week for DBP. Our term of interest is *ß*_4_, which reflects the difference in change in blood pressure per week per GI category, as compared to women in the lowest GI score quartile. Estimates and P values from repeated measurement models are given in Supplementary Table S2

**Fig. 3:**
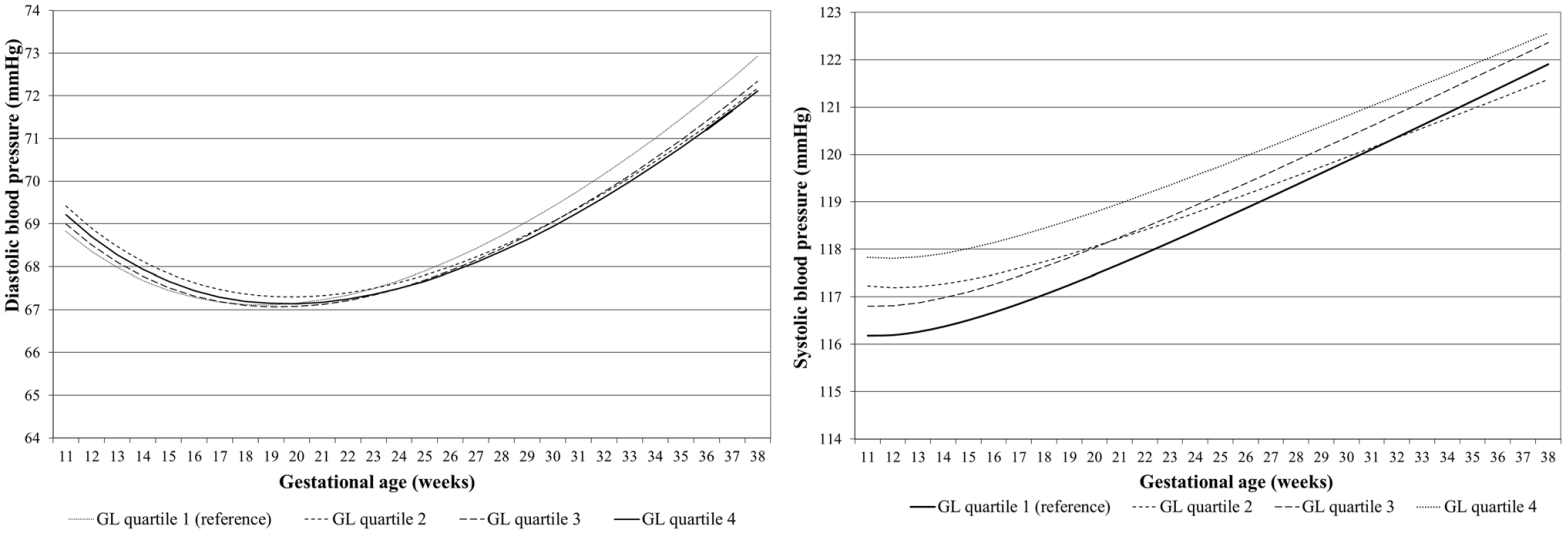
Blood pressure patterns in different glycemic load (GL) quartiles from repeated measurement models. Change in SBP and DBP in mmHg for first quartile, second quartile, third quartile and fourth quartile. SBP = *ß*_0_ + *ß*_1_ × GI quartile + *ß*_2_ × gestational age + *ß*_3_ × gestational age^−2^ + *ß*_4_ × GL quartile × gestational age. DBP = *ß*_0_ + *ß*_1_ × GL quartile + *ß*_2_ × gestational age + *ß*_3_ × gestational age^0.5^ + *ß*_4_ × GL quartile x gestational age. In these models, ‘*ß*_0_ + *ß*_1_ × GL’ reflects the intercept and ‘*ß*_2_ × gestational age + *ß*_3_ × gestational age^−2^ ‘reflects the slope of change in blood pressure per week for SBP, and ‘*ß*_2_ × gestational age + *ß*_3_ × gestational age^0.5^’, reflects the slope of change in blood pressure per week for DBP. Our term of interest is *ß*_4_, which reflects the difference in change in blood pressure per week per GL category, as compared to women in the lowest GL score quartile. Estimates and *P* values from repeated measurement models are given in Supplementary Table S2

Figure 2 shows the longitudinal systolic and diastolic blood pressure patterns throughout pregnancy per dietary glycemic index quartile. Women in the lowest dietary glycemic index quartile had the lowest systolic and diastolic blood pressure throughout pregnancy when compared to the other quartiles, although there were no significant differences in the increase of blood pressure per week present between quartiles (*p* values for interaction of dietary glycemic index quartile with gestational age ≥ 0.05). Similarly, no significant associations of dietary glycemic load quartiles with longitudinal blood pressure development throughout pregnancy were present (*p* values for interaction of dietary glycemic index quartile with gestational age ≥ 0.05) (Fig. 3). The regression coefficients for a gestational age-dependent and a gestational age-independent effect for dietary glycemic index and load quartiles are shown in Supplementary Table S2.

Table 2 shows that a higher dietary glycemic index and load across the full range were associated with a higher early-, mid- and late-pregnancy systolic blood pressure in the basic model, but these association disappeared after adjustment for socio-demographic factors. A higher dietary glycemic load across the full range was associated with a higher early-pregnancy diastolic blood pressure, which persisted after full adjustment for socio-demographic and lifestyle factors (0.98 (95% CI 0.35–1.61) mmHg per SDS increase in glycemic load). The effect estimate only partly attenuated but remained significant after additional adjustment for gestational weight gain, salt intake and dietary fiber intake (0.84 (95% CI 0.20, 1.50) mmHg per SDS increase in glycemic load). No other associations of dietary glycemic index and load across the full range with diastolic blood pressure were present. Dietary glycemic index and load quartiles were not associated with systolic and diastolic blood pressure in the fully adjusted models (Supplementary Table S3A-B).

**Table 2:** Associations of dietary glycemic index and glycemic load with systolic and diastolic blood pressure during pregnancy in total population (*n* = 3375)^a^

Glycemic index (SDS)	Differences in systolic blood pressure in mmHg (95% CI)		
	Early-pregnancy (*n* = 2802)	Mid-pregnancy (*n* = 3263)	Late-pregnancy (*n* = 3286)
Basic models^b^	0.38 (− 0.06, 0.82)	0.51 (0.11, 0.91)*	0.58 (0.19, 0.97)*
Socio-demographic models^c^	0.14 (− 0.31, 0.58)	0.20 (− 0.20, 0.61)	0.30 (− 0.09, 0.70)
Lifestyle models^d^	− 0.04 (− 0.46, 0.38)	0.03 (− 0.36, 0.41)	0.15 (− 0.24, 0.53)

	Differences in diastolic blood pressure in mmHg (95% CI)		
	Early-pregnancy (*n* = 2802)	Mid-pregnancy (*n* = 3262)	Late-pregnancy (*n* = 3285)
Basic models^b^	0.31 (− 0.03, 0.65)	0.41 (0.09, 0.72)*	0.26 (− 0.05, 0.58)
Socio-demographic models^c^	0.25 (− 0.10, 0.60)	0.29 (− 0.3, 0.61)	0.18 (− 0.14, 0.49)
Lifestyle models^d^	0.21 (− 0.12, 0.54)	0.21 (− 0.09, 0.52)	0.13 (− 0.17, 0.43)

Glycemic load (SDS)	Differences in systolic blood pressure in mmHg (95% CI)		
	Early-pregnancy (*n* = 2802)	Mid-pregnancy (*n* = 3263)	Late-pregnancy (*n* = 3286)
Basic model^b^	0.81 (0.37, 1.25)*	0.40 (− 0.01, 0.80)	0.47 (0.08, 0.86)*
Socio-demographic models^c^	0.77 (0.33, 1.20)*	0.33 (− 0.07, 0.73)	0.42 (0.03, 0.80)*
Lifestyle models^d^	0.23 (− 0.59, 1.05)	− 0.10 (− 0.84, 0.64)	0.05 (− 0.68, 0.78)

	Differences in diastolic blood pressure in mmHg (95% CI)		
	Early-pregnancy (*n* = 2802)	Mid-pregnancy (*n* = 3262)	Late-pregnancy (*n* = 3285)
Basic models^b^	0.35 (0.02, 0.69)*	0.06 (− 0.26, 0.38)	− 0.03 (− 0.35, 0.28)
Socio-demographic models^c^	0.35 (0.01, 0.69)*	0.05 ( −0.27, 0.36)	− 0.04 (− 0.35, 0.27)
Lifestyle models^d^	0.98 (0.35, 1.61)*	0.30 (− 0.28, 0.88)	0.27 (− 0.31, 0.84)

*SDS* standard deviation score. *CI* Confidence Interval

*P value < 0.05

^a^Values are regression coefficients (95% confidence interval) from multiple linear regression models and reflect the differences in mmHg blood pressure per one increase in standard deviation score of maternal glycemic index and glycemic load. Estimates are from multiple imputed data

^b^Basic models are adjusted for gestational age at time of intake

^c^Socio-demographic models are adjusted for maternal age, educational level, parity and gestational age at time of measurements

^d^Lifestyle models are adjusted for maternal age, educational level, parity, prepregnancy BMI, kcal, smoking habits, alcohol use, folic acid use and gestational age at time of the measurements

### Dietary glycemic index and load with placental vascular function

Table 3 shows that no consistent associations of dietary glycemic index and load across the full range with UmPI, UtRI and bilateral uterine artery notching were present after considering other maternal socio-demographic and lifestyle characteristics. A higher glycemic load was only associated with a lower UtRI (*p* value < 0.05). This association remained present after additional adjustment for dietary factors. No associations of dietary glycemic index and load quartiles with placental hemodynamic parameters were present (Supplementary Table S4A-B).

**Table 3:** Associations of dietary glycemic index and glycemic load with uterine artery resistance index, umbilical artery pulsatility index and bilateral uterine artery notching in total population (*n* = 3090)^a^

	Differences in UmPI (95% CI)^a^		Differences in UtRI (95% CI)^a^		Bilateral uterine artery notching (95% CI)^b^
	Mid-pregnancy *n* = 2505	Late-pregnancy *n* = 2751	Mid-pregnancy *n* = 1884	Late-pregnancy *n* = 2060	Late-pregnancy *n*_cases_ = 48
Glycemic index (SDS)					
Basic models^c^	− 0.001 (− 0.008, 0.007)	0.007 (0.000, 0.013)	− 0.004 (− 0.008, 0.001)	− 0.001 (− 0.004, 0.003)	1.12 (0.84, 1.49)
Socio-demographic models^d^	− 0.002 (− 0.009, 0.005)	0.005 (− 0.001, 0.011)	− 0.004 (− 0.008, 0.001)	− 0.001 (− 0.004, 0.003)	1.10 (0.95, 1.28)
Lifestyle models^e^	− 0.003 (− 0.011, 0.004)	0.004 (− 0.002, 0.011)	− 0.004 (− 0.008, 0.000)	− 0.001 (− 0.005, 0.002)	1.10 (0.82, 1.48)
Glycemic load (SDS)					
Basic models^c^	− 0.003 (− 0.010, 0.004)	0.002 (− 0.004, 0.008)	0.000 (− 0.004, 0.005)	0.000 (0.003, 0.004)	0.99 (0.74, 1.33)
Socio-demographic models^d^	− 0.003 (− 0.010, 0.004)	0.002 (− 0.004, 0.008)	0.000 (− 0.004, 0.005)	0.001 (− 0.001, 0.002)	0.98 (0.84, 1.13)
Lifestyle models^e^	− 0.011 (− 0.025, 0.003)	0.007 (− 0.005, 0.020)	− 0.004 (− 0.012, 0.004)	− 0.009 (− 0.015, − 0.002)*	1.05 (0.59, 1.84)

*UmPI* umbilical artery pulsatility index. *UtRI* uterine artery pulsatility index. *SDS* standard deviation score. *CI* Confidence Interval

*P value < 0.05

^a^Values are regression coefficients (95% confidence interval) from multiple linear regression models and reflect the differences in umbilical artery pulsatility index and uterine artery resistance index per one increase in standard deviation score of maternal glycemic index and glycemic load. Estimates are from multiple imputed data

^b^Values are odds ratios (95% confidence interval) from multiple logistic regression models and reflect the difference in risks of bilateral uterine artery notching per one increase in standard deviation score of maternal glycemic index and load. Estimates are from multiple imputed data

^c^Basic models are adjusted for gestational age at time of intake

^d^Socio-demographic models are adjusted for maternal age, educational level, parity and gestational age at time of measurements

^e^Lifestyle models are adjusted for maternal age, educational level, parity, prepregnancy BMI, kcal, smoking habits, alcohol use, folic acid use and gestational age at time of the measurements

### Maternal glycemic index and load and risks of gestational hypertensive disorders

Table 4 shows that dietary glycemic index and load across the full range were not associated with the risk of any gestational hypertensive disorder in the basic or adjusted models. No associations of dietary glycemic index and load quartiles with gestational hypertensive disorders were present (Supplementary Table S5A-B).

**Table 4:** Associations of dietary glycemic index and glycemic load with hypertensive disorder of pregnancy, gestational hypertension and preeclampsia in total population (*n* = 3299)^a^

	Gestational hypertensive disorders^b^	Gestational hypertension^b^	Preeclampsia^b^
	OR (95% CI) *n*_cases_ = 224	OR (95% CI) *n*_cases_ = 166	OR (95% CI) *n*_cases_ = 58
Glycemic index (SDS)			
Basic models^b^	1.00 (0.87, 1.14)	1.02 (0.87, 1.19)	0.92 (0.71, 1.20)
Socio-demographic models^c^	0.99 (0.86, 1.14)	1.01 (0.86, 1.19)	0.94 (0.72, 1.23)
Lifestyle models^d^	0.98 (0.85, 1.13)	1.00 (0.85, 1.19)	0.92 (0.70, 1.21)
Glycemic load (SDS)			
Basic model^b^	1.04 (0.91, 1.19)	1.03 (0.88, 1.20)	1.06 (0.83, 1.37)
Socio-demographic models^c^	1.05 (0.92, 1.21)	1.04 (0.89, 1.22)	1.10 (0.85, 1.42)
Lifestyle models^d^	0.98 (0.75, 1.30)	1.02 (0.75, 1.41)	0.89 (0.53, 1.49)

*SDS* standard deviation score; *CI* Confidence Interval

*P value < 0.05

^a^Values are odds ratios (95% confidence interval) from multiple logistic regression models and reflect the difference in risks of gestational hypertensive disorders, gestational hypertension and preeclampsia per one increase in standard deviation score of maternal glycemic index and glycemic load. Estimates are from multiple imputed data

^b^Basic models are adjusted for gestational age at time of intake

^c^Socio-demographic models are adjusted for maternal age, educational level, parity and gestational age at time of intake

^d^Lifestyle models are adjusted for maternal age, educational level, parity, prepregnancy BMI, kcal, smoking habits, alcohol use, folic acid use and gestational age at time of intake

### Sensitivity analyses

No associations were present with blood pressure, placental vascular function and gestational hypertensive disorders when we repeated the analyses using clinical cut-offs to classify glycemic index diets (Supplementary Table 6A–C). When we restricted our analyses to women with a BMI ≥ 25, a higher dietary glycemic index across the full range was only associated with a higher late pregnancy UmPI in all models (*p* value < 0.05) (Supplementary Table S7A–C). When we restricted to women who enrolled in the study before 14 weeks of gestation, no consistent associations with blood pressure, placental hemodynamic parameters and risk of gestational hypertensive disorders were present (Supplementary Table S8A–C). When we restricted to women with complete data on all covariates, we observed similar findings as in the main analyses (Supplementary Table S9A–C).

## Discussion

In this prospective cohort study we observed that dietary glycemic index and load during pregnancy were not consistently associated with blood pressure throughout pregnancy, placental vascular function or the risk of gestational hypertensive disorders after considering other maternal socio-demographic and lifestyle characteristics. Higher dietary glycemic load across the full range was only associated with a higher diastolic blood pressure in early-pregnancy.

### Interpretation of main findings

There is an increasing interest in low-glycemic index and load diets as a lifestyle intervention during pregnancy to improve birth outcomes [26]. In this low-risk pregnant population we observed that dietary glycemic index and load during pregnancy were not consistently associated with blood pressure and placental vascular function throughout pregnancy when also considering other socio-demographic and lifestyle factors. We only observed that a higher dietary glycemic load was associated with a higher early-pregnancy diastolic blood pressure after adjustment for socio-demographic, lifestyle and other dietary factors, but the effect estimate was only small. To our knowledge, we are the first study to investigate the associations of dietary glycemic index and load with blood pressure and placental vascular function during pregnancy. A meta-analysis of randomized controlled trials among 1097 healthy non-pregnant individuals indicated that a lower glycemic index or load diet is associated with a lower systolic and diastolic blood pressure [3]. The observed differences between this meta-analysis and our study may be explained by the overrepresentation of participants at high-risk of impaired glucose metabolism due to adiposity in the trials included in the meta-analysis and a greater magnitude of change in dietary glycemic index and load in the included intervention trials. As many of the studies also aimed to achieve weight reduction, it is hard to isolate the effect on blood pressure alone and to make the comparison with a pregnant population [3]. Finally, physiological changes related to pregnancy may further complicate the comparison of our results among a pregnant population to this meta-analyses among non-pregnant populations. During pregnancy a physiological decrease in systemic vascular resistance results in an initial decrease in blood pressure levels and physiologic metabolic adaptations during pregnancy lead to increased insulin resistance [39]. In our study, we observed no associations of dietary glycemic index and load with blood pressure in overweight or obese pregnant women, but a higher dietary glycemic index was associated with a higher umbilical artery pulsatility index in late-pregnancy only. Possibly, different effects of dietary glycemic index and load on vascular function might be present among pregnant women, as pregnancy related adaptations in the cardiovascular system occur. It could be hypothesized that the effects on endothelial function are most apparent in the fetoplacental vasculature as the vasomotor tone is completely driven by endothelial derived mediators [40–42]. Pregnancy related insulin resistance and subsequent effect on the endothelium will be more apparent in late-pregnancy, especially in overweight women. Although we did not observe consistent associations of maternal dietary glycemic index and load with gestational hemodynamic adaptations in our low-risk population, possible effects of the dietary glycemic index and load on gestational hemodynamic adaptations may be more pronounced among higher risk populations.

Only two studies examined the effects of carbohydrate quality on the risk of gestational hypertension and preeclampsia. A case–control study in Iran among 202 pregnant women showed a lower incidence of gestational hypertension when women consumed a below average daily glycemic load, but no associations were found for the glycemic index [21]. Within this Iranian study, recall and observer bias could be an issue as dietary intake of the previous year was assessed by a dietitian after the 20th week of pregnancy once gestational hypertension was already diagnosed and only prepregnancy BMI, age and education were considered as confounding factors [21]. Second, an intervention study in Italy among 370 overweight pregnant women found a lower incidence of gestational hypertension among women prescribed a customized low-glycemic index diet with physical activity counseling according to the ACOG and ACSM recommendations [22, 43]. We observed no effects of dietary glycemic index and load on the risk of gestational hypertensive disorders. The different findings can be explained as our study population reflects a low-risk population and we were able to correct for more confounding factors in our statistical analysis.

Within our low-risk Dutch population, we observed no consistent associations of dietary glycemic index and load with hemodynamic adaptations and the risk of gestational hypertensive disorders. Our study population reflects a relatively healthy pregnant population at low-risk for impaired glucose metabolism and at low-risk for gestational hypertensive disorders as we excluded women with diabetes and preexistent hypertension. Also among overweight and obese women, who are at higher risk for impaired glucose metabolism, we did not find consistent associations. Possibly, the beneficial effects of a lower dietary glycemic index and load on gestational hemodynamic adaptations are only apparent in diabetic women with profound impaired glucose metabolism who are at high risk of developing gestational hypertensive disorders. As we only had a small number of women with diabetes and gestational diabetes, we were not able to assess these associations. Furthermore, the dietary glycemic index and load within our study population were within a normal range, when compared to classification used for individual food products. Effects on gestational hemodynamic adaptations might only be present when larger differences from a higher dietary glycemic index and load to a lower dietary-glycemic index and load are achieved. The FFQ assessment in our study mainly reflected dietary intake in preconception period and the first trimester of pregnancy, which allowed us to investigate the association of dietary glycemic index and load on hemodynamic adaptations from early pregnancy onwards. Importantly, pregnancy related insulin resistance increases from mid-pregnancy onwards and effects of dietary glycemic index and load may be more pronounced in the second half of pregnancy.

## Strengths and limitations

The prospective data collection from early-pregnancy onwards with repeatedly measured blood pressure and placental hemodynamic parameters within a large study sample are major strengths of our study. The overall response rate for participation in the Generation R study was 61% and the participation in the self-administrated FFQs was 78% [24]. As we restricted to a Dutch population, this may have affected the generalizability of our findings. Furthermore, we had a relatively small number of gestational hypertensive disorder cases which indicates a possible selection towards a relatively healthy population. This relatively low number of cases might have caused a decreased statistical power for the gestational hypertensive disorder analyses. Studies in higher-risk population with more cases of preeclampsia and gestational hypertension are needed to examine these associations further. The FFQ is a widely used method to assess dietary intake in large observational studies, but relies on self-reported data which may be prone to over- or underreporting of dietary intake. Although the FFQ was not directly validated for the estimation of dietary glycemic index and load, the FFQ was shown to be a reliable tool for the estimation of total carbohydrate intake in a validation study conducted in the same area as the study area [25]. Within this validation study using 24 h dietary recalls and nutritional biomarkers, intake of carbohydrate was only slightly underestimated with the use of the FFQ [25]. When compared to the general Dutch population, we observed only a slightly lower mean maternal early-pregnancy dietary glycemic index [27]. This might be explained by slight underreporting of carbohydrate containing food products or may reflect our relatively healthier study population. The mean dietary glycemic index within our study was in line with the mean dietary glycemic index in other observational studies during pregnancy which are comparable in demographic and other lifestyle characteristics [28, 29]. We examined the associations of maternal dietary glycemic index and load with multiple outcomes, which might increase the risk of chance findings due to multiple testing. We did not perform correction for multiple testing as the evaluated outcome measures are strongly correlated. The observed associations of dietary glycemic load with early-pregnancy diastolic blood pressure among the total study population and dietary glycemic index with late-pregnancy umbilical artery pulsatility index among overweight and obese women, should be considered hypothesis generating and need further replication. Lastly, it is well-known that dietary intake is strongly related to socio-demographic and lifestyle factors. Detailed information about a large number of maternal sociodemographic and lifestyle factors was available within our study. Residual confounding might still be an issue because of the observational study design, for example by physical activity.

## Conclusion

Within a low-risk pregnant population, we did not find consistent associations of dietary glycemic index and load with blood pressure throughout pregnancy, placental vascular function and the risk of gestational hypertensive disorders. Further studies should focus on the effects of dietary glycemic index and load on gestational hemodynamic adaptations and the risk of gestational hypertensive disorders within pregnant populations at higher risk of impaired glucose metabolism.

## Supplementary Information

Below is the link to the electronic supplementary material.

Supplementary file1 (PDF 518 KB)

## Data Availability

Data is available with the authors up on reasonable request.

## Abbreviations

BMI: Body Mass Index
CI: Confidence interval
FFQ: Food frequency questionnaire
GI: Glycemic index
GL: Glycemic load
SD: Standard deviation
SDS: Standard deviation score
UmPI: Umbilical artery pulsatility index
UtRI: Uterine artery resistance index
