# Supplementary material for: Associations of dietary glycemic index and load during pregnancy with blood pressure, placental hemodynamic parameters and the risk of gestational hypertensive disorders
Source: Eur J Nutr. 2021 Sep 15;61(2):703–16. doi: 10.1007/s00394-021-02670-5 (PMC8854313; doi:10.1007/s00394-021-02670-5)
Supplement: Supplementary file 1 — Supplementary file1 (PDF 518 KB) [file 394_2021_2670_MOESM1_ESM.pdf]

Supplementary material

Associations of dietary glycemic index and load during pregnancy with blood pressure, placental hemodynamic parameters and the risk of gestational hypertensive disorders.

Running title: Dietary glycemic index and load with gestational hypertensive disorders

Clarissa J. Wiertsema MD[1,2], Rama J. Wahab MD[1,2], Annemarie G.M.G.J. Mulders MD PhD[3], Romy Gaillard MD PhD[1,2].

Author Affiliations:
1. The Generation R Study Group, Erasmus MC, University Medical Center, Rotterdam, the Netherlands.
2. Department of Pediatrics, Sophia's Children's Hospital, Erasmus MC, University Medical Center, Rotterdam, the Netherlands.
3. Departments of Obstetrics and Gynecology, Erasmus University Medical Center, Rotterdam, The Netherlands.

Corresponding author
Romy Gaillard, The Generation R Study Group, Erasmus University Medical Center, PO Box 2040, 3000 CA Rotterdam, the Netherlands. Email: r.gaillard@erasmusmc.nl. Telephone number: 0031 10 704 3405.

INDEX SUPPLEMENTAL MATERIAL

Page		
3	Table S1	Non-response analysis: characteristics of the total study population versus participating women without data on dietary intake
4	Figure S1	Directed Acyclic Graph for initial confounder selection.
5	Table S2	Longitudinal associations of glycemic index and load with systolic and diastolic blood pressure[a]
6	Table S3	A) Basic models: Associations of dietary glycemic index and load quartiles with systolic and diastolic blood pressure during pregnancy in total population<br>B) Confounder models: Associations of dietary glycemic index and load quartiles with systolic and diastolic blood pressure during pregnancy in total population
8	Table S4	A) Basic models: Associations of dietary glycemic index and load quartiles with umbilical artery pulsatility index, uterine artery resistance index and bilateral notching in total population<br>B) Confounder models: Associations of dietary glycemic index and load quartiles with umbilical artery pulsatility index, uterine artery resistance index and bilateral notching in total population
10	Table S5	A) Basic models: Associations of dietary glycemic index and load quartiles with hypertensive disorder of pregnancy, gestational hypertension and preeclampsia in total population<br>B) Confounder models: Associations of dietary glycemic index and load quartiles with hypertensive disorder of pregnancy, gestational hypertension and preeclampsia in total population
12	Table S6	A) Sensitivity analysis: Associations of dietary glycemic index with clinical cut-off for low- and normal-glycemic diet with systolic and diastolic blood pressure during pregnancy<br>B) Sensitivity analysis: Associations of dietary glycemic index with clinical cut-off for low- and normal-glycemic diet with umbilical artery pulsatility index, uterine artery resistance index and bilateral notching<br>C) Sensitivity analysis: Associations of dietary glycemic index with clinical cut-off for low and normal glycemic diet with hypertensive disorder of pregnancy, gestational hypertension and preeclampsia

15	Table S7	A) Sensitivity analysis: Associations of dietary glycemic index and load with systolic and diastolic blood pressure during pregnancy in population with BMI≥25<br>B) Sensitivity analysis: Associations of dietary glycemic index and load with umbilical artery pulsatility index, uterine artery resistance index and bilateral notching in population with BMI≥25<br>C) Sensitivity analysis: Associations of dietary glycemic index and load with hypertensive disorder of pregnancy, gestational hypertension and preeclampsia in population with BMI≥25
18	Table S8	A) Sensitivity analysis: Associations of dietary glycemic index and load with systolic and diastolic blood pressure during pregnancy in population with study enrollment <14 weeks of gestation<br>B) Sensitivity analysis: Associations of dietary glycemic index and load with uterine artery resistance index, umbilical artery pulsatility index and bilateral notching in population with study enrollment <14 weeks of gestation<br>C) Sensitivity analysis: Associations of dietary glycemic index and load with hypertensive disorder of pregnancy, gestational hypertension and preeclampsia in population with study enrollment <14 weeks of gestation
21	Table S9	A) Sensitivity analysis: Associations of dietary glycemic index and glycemic load with systolic and diastolic blood pressure during pregnancy in complete cases<br>B) Sensitivity analysis: Associations of dietary glycemic index and glycemic load with uterine artery resistance index, umbilical artery pulsatility index and bilateral uterine artery notching in complete cases<br>C) Sensitivity analysis: Associations of dietary glycemic index and glycemic load with hypertensive disorder of pregnancy, gestational hypertension and preeclampsia in complete cases

Supplementary Table S1. Non-response analysis: characteristics of the total study population versus participating women without data on dietary intake

	**Total group**	**Participants without data on dietary intake**[a]	
	n=3,378	n=506	p-value[b]
Maternal age at enrolment, years	31.4 (4.4)	30.2 (5.3)	<0.001
Gestational age at intake, weeks	13.5 (5.4-38.1)	14.1 (10.3-30.4)	<0.001
Parity, n nulliparous	2019 (59.9)	290 (57.8)	0.27
Prepregnancy BMI, kg/m^2	23.1 (3.8)	23.1 (4.1)	0.12
Prepregnancy BMI ≥25, n	636 (21.8)	96 (22.9)	
Gestational weigh gain, kg	10.8 (4.4)	11.2 (4.9)	0.06
Education, n high	1985 (59.5)	229 (46.5)	<0.001
Smoking, n continued during pregnancy	531 (17.0)	116 (25.7)	<0.001
Alcohol, n continued during pregnancy	1559 (50.2)	200 (44.5)	0.03
Early-pregnancy, ≥1 glass per week	830 (26.9)	98 (21.9)	0.03
Mid-pregnancy, ≥1 glass per week	378 (12.3)	34 (8.9)	0.06
Late-pregnancy ≥1 glass per week	444 (14.7)	45 (11.4)	0.08
Folic acid supplement use, n yes	2467 (89.1)	328 (82.2)	<0.001
Systolic blood pressure, mmHg			
Early-pregnancy	117.1 (11.8)	117.7 (12.2)	0.83
Mid-pregnancy	118.4 (11.7)	118.6 (10.9)	0.14
Late-pregnancy	120.3 (11.4)	119.7 (11.4)	0.75
Diastolic blood pressure, mmHg			
Early-pregnancy	68.4 (9.2)	68.0 (11.4)	0.88
Mid-pregnancy	67.1 (9.2)	66.0 (9.4)	0.29
Late-pregnancy	69.3 (9.1)	68.0 (9.3)	0.70
Uterine artery resistance index			
Mid-pregnancy	0.535 (0.089)	0.545 (0.090)	0.72
Late-pregnancy	0.483 (0.078)	0.480 (0.077)	0.72
Umbilical artery pulsatility index			
Mid-pregnancy	1.188 (0.183)	1.216 (0.180)	0.85
Late-pregnancy	0.977 (0.166)	0.985 (0.180)	0.07
Late-pregnanccy uterine artery notching	48 (2.2)	2 (0.7)	0.07
Gestational hypertensive disorders			
Gestational hypertension	166 (5.1)	24 (5.0)	0.74
Preeclampsia	58 (1.9)	8 (1.7)	0.80

Values are means (SD), median (95% range) or n (valid %). [a]Women of Dutch ethnicity enrolled during pregnancy with singleton live births without data on dietary intake. [b]p-values were obtained independent t-test or Mann-Whitney U-test for continuous variables and chi-square tests for categorical variables.

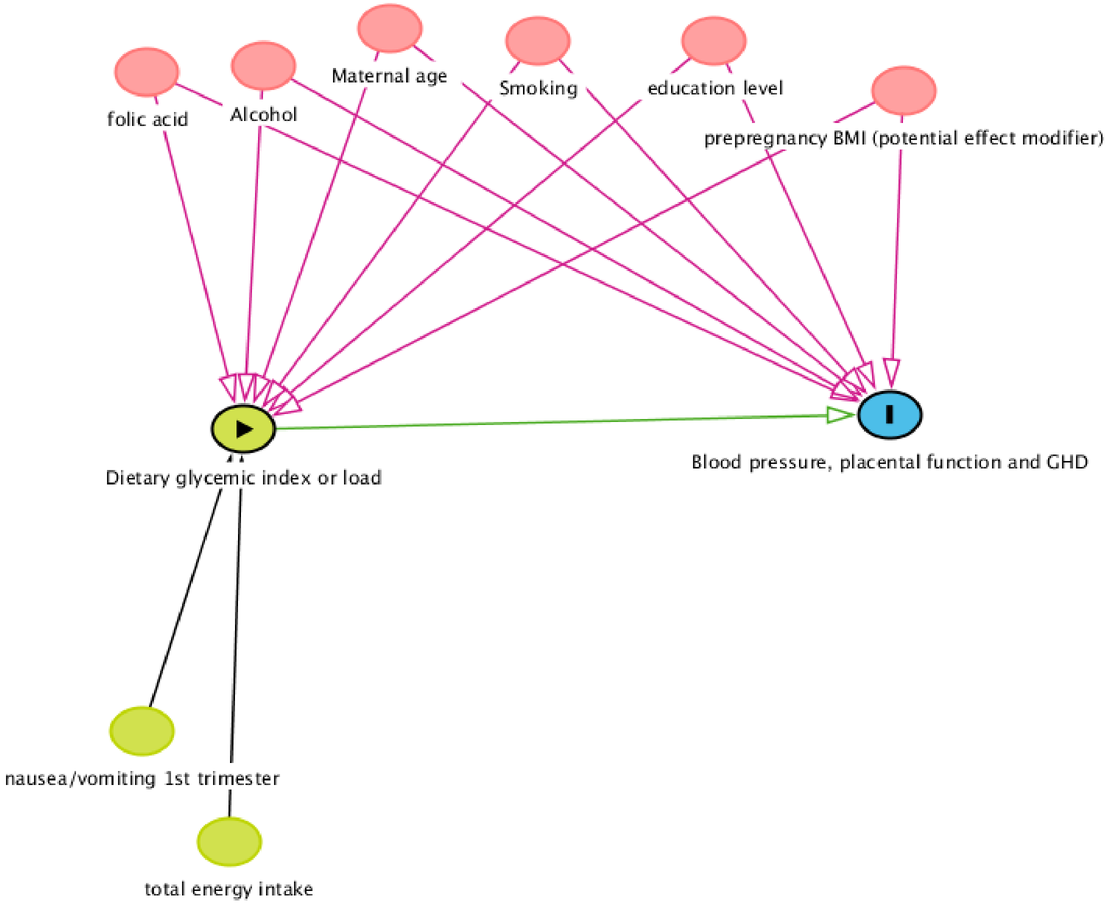

Supplementary Figure S2. Directed Acyclic Graph for confounder selection.

Supplementary Table S2. Longitudinal associations of glycemic index and load with systolic and diastolic blood pressure from repeated measurement models[a]

	**Systolic blood pressure in mmHg**			
	Intercept	p-value[b]	Slope (mmHg(95%CI))	p-value[b]
GI quartile 1	111.89	Reference	Reference	Reference
GI quartile 2	112.64	0.40	0.02	0.64
GI quartile 3	113.88	0.03	-0.01	0.83
GI quartile 4	112.68	0.38	0.04	0.25
GL quartile 1	111.66	<0.001	Reference	
GL quartile 2	113.27	0.07	-0.05	0.14
GL quartile 3	112.36	0.43	-0.01	0.85
GL quartile 4	113.72	0.02	-0.04	0.29
	**Diastolic blood pressure in mmHg**			
	Intercept	p-value[b]	Slope (mmHg(95%CI))	p-value[b]
GI quartile 1	99.24	Reference	Reference	Reference
GI quartile 2	98.99	0.72	0.03	0.26
GI quartile 3	100.08	0.23	0.002	0.93
GI quartile 4	99.75	0.47	0.02	0.34
GL quartile 1	98.96	Reference	Reference	Reference
GL quartile 2	100.10	0.10	-0.04	0.06
GL quartile 3	99.45	0.48	-0.03	0.29
GL quartile 4	99.83	0.21	-0.05	0.10

CI, Confidence interval. [a]Values are based on repeated non-linear regression models and reflect the change in blood pressure in mmHg per glycemic index and glycemic load quartile compared to women with the highest dietary quality (quartile 1) as reference. Models are adjusted for gestational age at the time of intake. [b]P-value reflects the significance level of the estimate.

Supplementary table 3a. Basic models: Associations of dietary glycemic index and load quartiles with systolic and diastolic blood pressure during pregnancy in total population (n=3,375)[a].

	**Differences in systolic blood pressure in mmHg (95% CI)**		
	Early-pregnancy[b] n=2,802	Mid-pregnancy[b] n=3,263	Late-pregnancy[b] n=3,286
GI quartile 1	reference	reference	reference
GI quartile 2	0.55 (-0.69, 1.80)	1.47 (0.34, 2.60)*	1.20 (0.10, 2.30)*
GI quartile 3	1.66 (0.42, 2.90)*	2.00 (0.87, 3.13)*	1.63 (0.53, 2.72)*
GI quartile 4	1.03 (-0.21, 2.26)	1.85 (0.72, 2.98)*	1.83 (0.73, 2.94)*
GL quartile 1	reference	reference	Reference
GL quartile 2	1.09 (-0.14, 2.36)	0.62 (-0.52, 1.75)	0.03 (-1.07, 1.13)
GL quartile 3	0.98 (-0.25, 2.20)	0.39 (-0.75, 1.52)	0.57 (-0.53, 1.67)
GL quartile 4	2.07 (0.84, 3.31)*	0.98 (-0.16, 2.11)	1.12 (0.02, 2.22)*
	**Differences in diastolic blood pressure in mmHg (95% CI)**		
	Early-pregnancy[b] n=2,802	Mid-pregnancy[b] n=3,262	Late-pregnancy[b] n=3,285
GI quartile 1	reference	reference	reference
GI quartile 2	0.17 (-0.79, 1.14)	0.38 (-0.52, 1.27)	0.67 (-0.23, 1.54)
GI quartile 3	0.58 (-0.38, 1.54)	1.36 (0.46, 2.25)*	0.63 (-0.25, 1.52)
GI quartile 4	0.83 (-0.13, 1.78)	1.29 (0.39, 2.18)*	0.96 (0.07, 1.84)*
GL quartile 1	reference	reference	reference
GL quartile 2	0.55 (-0.41, 1.50)	0.33 (-0.56, 1.23)	-0.47 (-1.36, 0.42)
GL quartile 3	0.27 (-0.68, 1.22)	0.10 (-0.80, 1.00)	-0.44 (-1.33, 0.44)
GL quartile 4	0.63 (-0.33, 1.58)	-0.08 (-0.98, 0.81)	-0.39 (-1.28, 0.49)

CI, Confidence Interval. GI, glycemic index. GL, glycemic load. [a]Values are regression coefficients (95% confidence interval) and reflect the difference in mmHg blood pressure per glycemic index or glycemic load quartile. Groups are compared to women with the highest dietary quality (quartile 1) as reference. Estimates are from multiple imputed data. [b]Models are adjusted for gestational age at time of intake. [c]Tests for trend were based on multiple linear regression models with glycemic index and load as z-score. *P-value <0.05.

Supplementary table 3b. Confounder model: Associations of dietary glycemic index and load quartiles with systolic and diastolic blood pressure during pregnancy in total population (n=3,375)[a].

	Differences in systolic blood pressure in mmHg (95% CI)		
	Early-pregnancy[b] n=2,802	Mid-pregnancy[b] n=3,263	Late-pregnancy[b] n=3,286
GI quartile 1	reference	reference	reference
GI quartile 2	-0.03 (-1.20, 1.14)	0.86 (-0.20, 1.92)	0.61 (-0.43, 1.66)
GI quartile 3	0.75 (-0.42, 1.92)	1.00 (-0.07, 2.07)	0.79 (-0.26, 1.84)
GI quartile 4	-0.22 (-1.41, 0.97)	0.47 (-0.63, 1.56)	0.56 (-0.52, 1.64)
GL quartile 1	Reference	reference	reference
GL quartile 2	0.67 (-0.63, 1.96)	0.39 (-0.79, 1.58)	0.11 (-1.05, 1.27)
GL quartile 3	-0.10 (-1.63, 1.43)	-0.50 (-1.89, 0.90)	0.07 (-1.30, 1.44)
GL quartile 4	0.30 (-1.70, 2.29)	-0.51 (-2.32, 1.31)	0.14 (-1.65, 1.92)
	**Differences in diastolic blood pressure in mmHg (95% CI)**		
	Early-pregnancy[b] n=2,802	Mid-pregnancy[b] n=3,262	Late-pregnancy[b] n=3,285
GI quartile 1	reference	reference	reference
GI quartile 2	-0.08 (-0.99, 0.82)	0.03 (-0.80, 0.86)	0.33 (-0.49, 1.15)
GI quartile 3	0.24 (-0.67, 1.14)	0.80 (-0.04, 1.64)	0.27 (-0.56, 1.10)
GI quartile 4	0.48 (-0.44, 1.40)	0.69 (-0.17, 1.54)	0.49 (-0.37, 1.34)
GL quartile 1	reference	Reference	reference
GL quartile 2	0.68 (-0.32, 1.68)	0.40 (-0.53, 1.33)	-0.15 (-1.07, 0.77)
GL quartile 3	0.48 (-0.71, 1.66)	-0.01 (-1.10, 1.09)	-0.25 (-1.33, 0.84)
GL quartile 4	0.99 (-0.55, 2.54)	-0.17 (-1.60, 1.25)	-0.15 (-1.56, 1.26)

CI, Confidence Interval. GI, glycemic index. GL, glycemic load. [a]Values are regression coefficients (95% confidence interval) and reflect the difference in mmHg blood pressure per glycemic index and glycemic load quartile. Groups are compared to women with the highest dietary quality (quartile 1) as reference. Estimates are from multiple imputed data. [b]Models are adjusted for maternal age, ethnicity, educational level, parity, prepregnancy BMI, kcal, smoking habits, alcohol use and gestational age at time of the measurements. *P-value <0.05.

Supplementary table 4a. Basic models: Associations of dietary glycemic index and load quartiles with umbilical artery pulsatility index, uterine artery resistance index and bilateral notching in total population (n=3,090)[a].

	**Differences in UmPI[a] (95% CI)**		**Differences in UtRI[a] (95% CI)**		**Bilateral notching[b] (95% CI)**
	Mid-pregnancy n=2,505	Late-pregnancy n=2,751	Mid-pregnancy n=1,884	Late-pregnancy n=2,060	Late-pregnancy n_{cases}=48
GI quartile 1	reference	reference	reference	reference	Reference
GI quartile 2	0.015 (-0.005, 0.035)	0.001 (-0.016, 0.019)	-0.008 (-0.019, 0.003)	-0.004 (-0.013, 0.006)	0.72 (0.33, 1.58)
GI quartile 3	0.018 (-0.002, 0.039)	0.011 (-0.006, 0.029)	-0.007 (-0.018, 0.004)	-0.005 (-0.014, 0.005)	0.41 (0.16, 1.06)
GI quartile 4	0.003 (-0.017, 0.023)	0.016 (-0.002, 0.033)	-0.007 (-0.018, 0.005)	-0.003 (-0.013, 0.006)	1.12 (0.55, 2.29)
GL quartile 1	reference	reference	reference	reference	reference
GL quartile 2	-0.002 (-0.022, 0.018)	-0.023 (-0.041, -0.006)*	-0.005 (-0.016, 0.006)	-0.003 (-0.012, 0.007)	0.99 (0.44, 2.23)
GL quartile 3	-0.001 (-0.021, 0.019)	-0.015 (-0.033, 0.002)	0.005 (-0.006, 0.016)	0.005 (-0.005, 0.014)	1.23 (0.57, 2.69)
GL quartile 4	-0.006 (-0.026, 0.015)	0.000 (-0.018, 0.018)	-0.003 (-0.015, 0.009)	-0.001 (-0.011, 0.008)	0.90 (0.39, 2.10)

UmPI, umbilical artery pulsatility index. UtRI, uterine artery resistance index. CI, Confidence Interval. GI, glycemic index. GL, glycemic load. [a]Values are regression coefficients (95% confidence interval) and reflect differences in umbilical artery pulsatility index and uterine artery resistance index per glycemic index and glycemic load quartile. Groups are compared to women with the highest dietary quality (quartile 1) as reference. [b]Values are odds ratios (95% confidence interval) that reflect difference in risks of bilateral notching per glycemic index an glycemic load quartile. Groups are compared to women with the highest dietary quality (quartile 1) as reference. Estimates are from multiple imputed data. [c]Models are adjusted for gestational age at time of intake. *P-value <0.05.

Supplementary table 4b. Confounder models: Associations of dietary glycemic index and load quartiles with umbilical artery pulsatility index, uterine artery resistance index and bilateral notching in total population (n=3,090)[a].

	**Differences in UmPI[a] (95% CI)**		**Differences in UtRI[a] (95% CI)**		**Bilateral notching[b] (95% CI)**
	Mid-pregnancy n=2,505	Late-pregnancy n=2,751	Mid-pregnancy n=1,884	Late-pregnancy n=2,060	Late-pregnancy n_{cases}=48
GI quartile 1	reference	reference	reference	reference	reference
GI quartile 2	0.011 (-0.009, 0.030)	0.000 (-0.017, 0.018)	-0.008 (-0.019, 0.003)	-0.003 (-0.013, 0.006)	0.71 (0.32, 1.53)
GI quartile 3	0.012 (-0.008, 0.032)	0.007 (-0.011, 0.024)	-0.007 (-0.018, 0.004)	-0.006 (-0.015, 0.004)	0.39 (0.15, 1.04)
GI quartile 4	-0.005 (-0.025, 0.015)	0.010 (-0.008, 0.028)	-0.008 (-0.019, 0.004)	-0.005 (-0.015, 0.005)	1.06 (0.53, 2.10)
GL quartile 1	reference	reference	reference	reference	reference
GL quartile 2	-0.001 (-0.023, 0.021)	-0.017 (-0.037, 0.002)	-0.008 (-0.020, 0.005)	-0.009 (-0.019, 0.001)	1.05 (0.42, 2.59)
GL quartile 3	-0.006 (-0.032, 0.020)	-0.012 (-0.035, 0.011)	0.000 (-0.015, 0.015)	-0.007 (-0.020, 0.005)	1.33 (0.46, 3.87)
GL quartile 4	-0.014 (-0.047, 0.020)	0.003 (-0.027, 0.032)	-0.011 (-0.030, 0.008)	-0.019 (-0.036, -0.003)*	0.98 (0.24, 4.04)

UmPI, umbilical artery pulsatility index. UtRI, uterine artery resistance index. CI, Confidence Interval. GI, glycemic index. GL, glycemic load. [a]Values are regression coefficients (95% confidence interval) and reflect differences in umbilical artery pulsatility index and uterine artery resistance index per glycemic index and glycemic load quartile. Groups are compared to women with the highest dietary quality (quartile 1) as reference. [b]Values are odds ratios (95% confidence interval) that reflect difference in risks of bilateral notching per glycemic index an glycemic load quartile. Groups are compared to women with the highest dietary quality (quartile 1) as reference. Estimates are from multiple imputed data. [c]Models are adjusted for maternal age, ethnicity, educational level, parity, prepregnancy BMI, kcal, smoking habits, alcohol use and gestational age at time of the measurements. *P-value <0.05.

Supplementary table 5a. Basic models: Associations of dietary glycemic index and load quartiles with hypertensive disorder of pregnancy, gestational hypertension and preeclampsia in total population (n=3,299)[a].

	**Gestational hypertensive disorders**[b]	**Gestational hypertension**[b]	**Preeclampsia**[b]
	Odds ratio (95% CI) n_{cases}=224	Odds ratio (95% CI) n_{cases}=166	Odds ratio (95% CI) n_{cases}=58
GI quartile 1	reference	reference	reference
GI quartile 2	1.03 (0.70, 1.52)	1.18 (0.76, 1.84)	0.69 (0.32, 1.49)
GI quartile 3	1.06 (0.72, 1.56)	1.03 (0.65, 1.63)	1.14 (0.58, 2.25)
GI quartile 4	1.07 (0.72, 1.57)	1.16 (0.75, 1.82)	0.83 (0.40, 1.74)
GL quartile 1	reference	reference	reference
GL quartile 2	1.06 (0.72, 1.56)	1.35 (0.86, 2.12)	0.50 (0.22, 1.11)
GL quartile 3	1.04 (0.71, 1.54)	1.18 (0.74, 1.87)	0.78 (0.39, 1.58)
GL quartile 4	1.15 (0.78, 1.68)	1.25 (0.79, 1.98)	0.95 (0.48, 1.85)

CI, Confidence Interval. GI, glycemic index. GL, glycemic load. [a]Values are odds ratios (95% confidence interval) that reflect difference in risks of Gestational hypertensive disorders, gestational hypertension and preeclampsia per glycemic index and glycemic load quartile. Groups are compared to women with the highest dietary quality (quartile 1) as reference. Estimates are from multiple imputed data. [b]Models are adjusted for gestational age at time of intake. *P-value <0.05.

Supplementary table 5b. Confounder models: Associations of dietary glycemic index and load quartiles with hypertensive disorder of pregnancy, gestational hypertension and preeclampsia in total population (n=3,299)[a].

	**Gestational hypertensive disorders[b]**	**Gestational hypertension[b]**	**Preeclampsia[b]**
	Odds ratio (95% CI) n_{cases}=224	Odds ratio (95% CI) n_{cases}=166	Odds ratio (95% CI) n_{cases}=58
GI quartile 1	reference	reference	reference
GI quartile 2	1.00 (0.67, 1.48)	1.12 (0.71, 1.76)	0.68 (0.31, 1.50)
GI quartile 3	0.97 (0.65, 1.45)	0.91 (0.57, 1.47)	1.09 (0.54, 2.21)
GI quartile 4	1.03 (0.68, 1.54)	1.10 (0.69, 1.77)	0.84 (0.39, 1.81)
GL quartile 1	reference	reference	reference
GL quartile 2	1.10 (0.70, 1.71)	1.50 (0.90, 2.51)	0.41 (0.17, 0.98)*
GL quartile 3	1.05 (0.63, 1.77)	1.35 (0.73, 2.48)	0.55 (0.21, 1.40)
GL quartile 4	1.14 (0.57, 2.24)	1.54 (0.70, 3.41)	0.51 (0.15, 1.78)

CI, Confidence Interval. GI, glycemic index. GL, glycemic load. [a]Values are odds ratios (95% confidence interval) that reflect difference in risks of Gestational hypertensive disorders, gestational hypertension and preeclampsia per glycemic index and glycemic load quartile. Groups are compared to women with the highest dietary quality (quartile 1) as reference. Estimates are from multiple imputed data. [b]Models are adjusted for maternal age, ethnicity, educational level, parity, prepregnancy BMI, kcal, smoking habits, alcohol use and gestational age at time of the measurements. *P-value <0.05.

Supplementary table 6a. Sensitivity analysis: Associations of dietary glycemic index with clinical cut-off for low- and normal-glycemic diet with systolic and diastolic blood pressure during pregnancy (n=3,374) [a].

		**Differences in systolic blood pressure in mmHg (CI 95%)**		
		Early-pregnancy	Mid-pregnancy	Late-pregnancy
Normal-GI		reference<br>n=1938	reference<br>n=2238	reference<br>n=2262
Low-GI	Basic model[b]	-0.92 (-1.87, 0.03)	-1.58 (-2.45, -0.72)*	-1.18 (-2.02, -0.34)*
	Lifestyle model[c]	-0.01 (-0.91, 0.90)<br>n=863	-0.63 (1.45, -1.19)<br>n=1024	-0.36 (-1.17, 0.45)<br>n=1023

		**Differences in diastolic blood pressure in mmHg (CI 95%)**		
		Early-pregnancy	Mid-pregnancy	Late-pregnancy
Normal-GI		reference<br>n=1938	reference<br>n=2237	reference<br>n=2261
Low-GI	Basic model[b]	-0.61 (-1.35, 0.12)	-1.03 (0.34, 1.71)*	-0.64 (-1.32, 0.03)
	Lifestyle model[c]	-0.27 (-0.97, 0.43)<br>n=863	-0.53 (-1.17, 0.12)<br>n=1024	-0.32 (-0.96, 0.32)<br>n=1023

GI, glycemic index. [a]Values are regression coefficients (95% confidence interval) and reflect the difference in mmHg blood pressure of a low-glycemic index diet compared to women with a normal-glycemic index diet as reference. Estimates are from multiple imputed data. [b]Basic models are adjusted for gestational age at time of intake. [c]Confounder models are adjusted for maternal age, educational level, parity, prepregnancy BMI, kcal, smoking habits, alcohol use, folic acid use and gestational age at time of the measurements. *P-value <0.05.

Supplementary table 6b. Sensitivity analysis: Associations of dietary glycemic index with clinical cut-off for low- and normal-glycemic diet with umbilical artery pulsatility index, uterine artery resistance index and bilateral notching (n=3,089) [a].

		Differences in UmPI (95% CI)[a]		Differences in UtRI (95% CI)[a]		Bilateral notching (95% CI)[b]
		Mid-pregnancy	Late-pregnancy	Mid-pregnancy	Late-pregnancy	Late-pregnancy
Normal-GI		reference<br>n=1,706	reference<br>n=1,897	reference<br>n=1,255	reference<br>n=1,409	reference<br>n_{cases}=30
Low-GI	Basic model[c]	-0.006 (-0.021, 0.009)	-0.006 (-0.007, 0.020)	0.006 (-0.003, 0.014)	0.005 (-0.002, 0.012)	1.32 (0.73, 2.39)
	Lifestyle model[d]	-0.001 (-0.017, 0.014)<br>n=799	-0.001 (-0.014, 0.013)<br>n=853	0.006 (-0.003, 0.014)<br>n=629	0.006 (-0.001, 0.013)<br>n=651	1.41 (0.77, 2.60)<br>n_{cases}=18

UmPI, umbilical artery pulsatility index. UtRI, uterine artery resistance index. CI, Confidence Interval. GI, glycemic index. [a]Values are regression coefficients (95% confidence interval) and reflect the differences in umbilical artery pulsatility index and uterine artery resistance index of women with a low-glycemic index diet compared to women with a normal-glycemic index diet as reference. Estimates are from multiple imputed data. [b]Values are odds ratios (95% confidence interval) from multiple logistic regression models and reflect the differences in risks of bilateral notching of women with a normal-glycemic index diet compared to women with a low-glycemic index diet as reference. Estimates are from multiple imputed data. [c]Basic models are adjusted for gestational age at time of intake. [d]Confounder models are adjusted for maternal age, educational level, parity, prepregnancy BMI, kcal, smoking habits, alcohol use, folic acid use and gestational age at time of the measurements. *P-value <0.05.

Supplementary table 6c. Sensitivity analysis: Associations of dietary glycemic index with clinical cut-off for low and normal glycemic diet with hypertensive disorder of pregnancy, gestational hypertension and preeclampsia (n=3,298) [a].

		**Gestational hypertensive disorders**	**Gestational hypertension**	**Preeclampsia**
		OR (95% CI)	OR (95% CI)	OR (95% CI)
Normal-GI<br>n=2,262		reference<br>n_{cases}=155	reference<br>n_{cases}=115	reference<br>n_{cases}=40
Low-GI<br>n=1,036	Basic model[b]	0.97 (0.73, 1.30)	0.97 (0.69, 1.36)	0.97 (0.56, 1.71)
	Lifestyle model[c]	1.03 (0.75, 1.40)<br>n_{cases}=69	1.05 (0.74, 1.50)<br>n_{cases}=51	0.98 (0.55, 1.74)<br>n_{cases}=18

OR, odds ratio. CI, Confidence Interval. GI, glycemic index. [a]Values are odds ratios (95% confidence interval) from multiple logistic regression models and reflect the difference in risks of gestational hypertensive disorders, gestational hypertension and preeclampsia of women with a low-glycemic index diet compared to women with a normal-glycemic index diet as reference. Estimates are from multiple imputed data. [b]Basic models are adjusted for gestational age at time of intake. [c]Confounder models are adjusted for maternal age, educational level, parity, prepregnancy BMI, kcal, smoking habits, alcohol use, folic acid use and gestational age at time of the measurements. *P-value <0.05.

Supplementary table 7a. Sensitivity analysis: Associations of dietary glycemic index and load with systolic and diastolic blood pressure during pregnancy in population with BMI≥25 (n=766) [a].

**Glycemic index (SDS)**	**Differences in systolic blood pressure in mmHg (CI 95%)**		
	Early-pregnancy (n=623)	Mid-pregnancy (n=728)	Late-pregnancy (n=741)
Basic models[b]	-0.22 (-1.23, 0.80)	-0.16 (-1.09, 0.76)	0.23 (-0.69, 1.16)
Socio-demographic models[c]	-0.39 (-1.36, 0.68)	-0.46 (-1.39, 0.47)	-0.08 (-1.01, 0.86)
Lifestyle models[e]	-0.32 (-1.32, 0.68)	-0.45 (-1.36, 0.46)	-0.18 (-1.09, 0.74)
	**Differences in diastolic blood pressure in mmHg (CI 95%)**		
	Early-pregnancy (n=623)	Mid-pregnancy (n=728)	Late-pregnancy (n=741)
Basic models[b]	-0.08 (-0.85, 0.69)	-0.27 (-0.99, 0.44)	-0.11 (-0.78, 0.57)
Socio-demographic models[c]	-0.09 (-0.87, 0.69)	-0.27 (-1.00, 0.47)	-0.06 (-0.75, 0.62)
Lifestyle models[e]	0.16 (-0.59, 0.92)	-0.12 (-0.81, 0.56)	0.09 (-0.57, 0.76)

**Glycemic load (SDS)**	**Differences in systolic blood pressure in mmHg (CI 95%)**		
	Early-pregnancy (n=623)	Mid-pregnancy (n=728)	Late-pregnancy (n=741)
Basic models[b]	1.11 (0.13, 2.08)*	0.25 (-0.66, 1.15)	0.79 (-0.06, 1.64)
Socio-demographic models[c]	0.98*(0.005, 1.96)*	0.07 (-0.82, 0.96)	0.64 (-0.19, 1.47)
Lifestyle models[e]	-0.10(-2.04, 1.84)	-1.29 (-3.00, 0.42)	-0.90 (-2.53, 0.74)
	**Differences in diastolic blood pressure in mmHg (CI 95%)**		
	Early-pregnancy (n=623)	Mid-pregnancy (n=728)	Late-pregnancy (n=741)
Basic models[b]	0.01 (-0.75, 0.77)	-0.32 (-1.02, 0.38)	-0.27 (-0.91, 0.37)
Socio-demographic models[c]	-0.05 (-0.82, 0.71)	-0.29 (-0.99, 0.41)	-0.29 (-0.93, 0.35)
Lifestyle models[e]	1.31 (-0.20, 2.82)	-0.28 (-1.56, 1.01)	0.43 (-0.82, 1.68)

SDS, standard deviation score. CI, Confidence Interval. [a]Values are regression coefficients (95% confidence interval) from multiple linear regression models and reflect the differences in mmHg blood pressure per one increase in standard deviation score of maternal glycemic index and glycemic load. Estimates are from multiple imputed data. [b]Basic models are adjusted for gestational age at time of intake. [c]Socio-demographic models are adjusted for maternal age, educational level, parity and gestational age at time of measurements. [d] Lifestyle models are adjusted for maternal age, educational level, parity, prepregnancy BMI, kcal, smoking habits, alcohol use, folic acid use and gestational age at time of the measurements. *P-value <0.05.

Supplementary table 7b. Sensitivity analysis: Associations of dietary glycemic index and load with umbilical artery pulsatility index, uterine artery resistance index and bilateral notching in population with BMI≥25 (n=766).

	**Differences in UmPI (95% CI)** [a]		**Differences in UtRI (95% CI)**[a]		**Bilateral notching (95% CI)**[b]
**Glycemic index (SDS)**	Mid-pregnancy n=547	Late-pregnancy n=609	Mid-pregnancy n=380	Late-pregnancy n=459	Late-pregnancy n_{cases}=11
Basic model[c]	-0.010 (-0.027, 0.007)	0.019 (0.005, 0.033)*	0.003 (-0.007, 0.013)	0.005 (-0.003, 0.012)	0.92 (0.44, 1.90)
Socio-demographic models[d]	-0.009 (-0.025, 0.008)	0.018 (0.004, 0.032)*	0.004 (-0.007, 0.014)	0.004 (-0.003, 0.012)	0.94 (0.46, 1.91)
Lifestyle models [e]	-0.012 (-0.029, 0.005)	0.016 (0.002, 0.030)*	0.003 (-0.008, 0.013)	0.004 (-0.004, 0.012)	0.86 (0.43, 1.83)
**Glycemic load (SDS)**	Mid-pregnancy n=547	Late-pregnancy n=609	Mid-pregnancy n=380	Late-pregnancy n=459	Late-pregnancy n_{cases}=11
Basic model[c]	-0.010 (-0.026, 0.007)	0.011 (-0.003, 0.025)	-0.001 (-0.011, 0.010)	0.005 (-0.002, 0.013)	0.81 (0.39, 1.66)
Socio-demographic models[d]	-0.007 (-0.022, 0.009)	0.011 (-0.003, 0.025)	0.001 (-0.010, 0.011)	0.006 (-0.002, 0.014)	0.80 (0.38, 1.68)
Lifestyle models [e]	-0.031 (-0.064, 0.002)	0.016 (-0.013, 0.044)	0.007 (-0.015, 0.029)	0.004 (-0.012, 0.020)	0.86 (0.17, 4.25)

SDS, standard deviation score. CI, Confidence Interval. UmPI, umbilical artery pulsatility index. UtRI, uterine artery resistance index. [a]Values are regression coefficients (95% confidence interval) from multiple linear regression models and reflect the differences in umbilical artery pulsatility index and uterine artery resistance index per one increase in standard deviation score of maternal glycemic index and glycemic load. Estimates are from multiple imputed data. [b]Values are odds ratios (95% confidence interval) from multiple logistic regression models and reflect the difference in risks of bilateral notching per one increase in standard deviation score of maternal glycemic index and load. Estimates are from multiple imputed data. [c]Basic models are adjusted for gestational age at time of intake. [d]Socio-demographic models are adjusted for maternal age, educational level, parity and gestational age at time of measurements. [e]Lifestyle models are adjusted for maternal age, educational level, parity, prepregnancy BMI, kcal, smoking habits, alcohol use, folic acid use and gestational age at time of the measurements.*P-value <0.05.

Supplementary table 7c. Sensitivity analysis: Associations of dietary glycemic index and load with hypertensive disorder of pregnancy, gestational hypertension and preeclampsia in population with BMI≥25 (n=766) [a].

	**Gestational hypertensive disorders**[b]	**Gestational hypertension**[b]	**Preeclampsia**[b]
**Glycemic index (SDS)**	Odds ratio(95% CI)	Odds ratio(95% CI)	Odds ratio(95% CI)
	n_{cases}=89	n_{cases}=74	n_{cases}=15
Basic model[c]	0.81 (0.63, 1.03)	0.71 (0.58, 0.98)*	1.08 (0.60, 1.94)
Socio-demographic models[d]	0.85 (0.66, 1.10)	0.80 (0.61, 1.06)	1.14 (0.61, 2.14)
Lifestyle models[e]	0.84 (0.64, 1.10)	0.80 (0.60, 1.07)	1.07 (0.53, 2.16)
**Glycemic load (SDS)**	Odds ratio(95% CI)	Odds ratio(95% CI)	Odds ratio(95% CI)
	n_{cases}=89	n_{cases}=74	n_{cases}=15
Basic model[c]	1.00 (0.80, 1.26)	0.95 (0.74, 1.22)	1.27 (0.77, 2.10)
Socio-demographic models[d]	1.04 (0.82, 1.32)	0.98 (0.76, 1.27)	1.34 (0.80, 2.24)
Lifestyle models[e]	0.95 (0.57, 1.59)	0.86 (0.50, 1.48)	1.38 (0.38, 5.03)

SDS, standard deviation score. CI, Confidence Interval. [b]Values are odds ratios (95% confidence interval) from multiple logistic regression models and reflect the difference in risks of gestational hypertensive disorders, gestational hypertension and preeclampsia per one increase in standard deviation score of maternal glycemic index and glycemic load. Estimates are from multiple imputed data. [c]Basic models are adjusted for gestational age at time of intake. [d]Socio-demographic models are adjusted for maternal age, educational level, parity and gestational age at time of intake. [e]Lifestyle models are adjusted for maternal age, educational level, parity, prepregnancy BMI, kcal, smoking habits, alcohol use, folic acid use and gestational age at time of intake. *P-value <0.05.

Supplementary table 8a. Sensitivity analysis: Associations of dietary glycemic index and load with systolic and diastolic blood pressure during pregnancy in population with study enrollment <14 weeks of gestation (n=1,867) [a].

Glycemic index (SDS)	Differences in systolic blood pressure in mmHg (CI 95%)		
	Early-pregnancy (n=1,848)	Mid-pregnancy (n=1,833)	Late-pregnancy (n=1,821)
Basic models[b]	0.12 (-0.43, 0.67)	0.34 (-0.21, 0.89)	0.32 (-0.22, 0.85)
Socio-demographic models[c]	-0.06 (-0.61, 0.50)	0.10 (-0.45, 0.65)	0.12 (-0.42, 0.65)
Lifestyle models[d]	-0.17 (-0.70, 0.36)	-0.02 (-0.54, 0.51)	0.06 (-0.46, 0.58)
	**Differences in diastolic blood pressure in mmHg (CI 95%)**		
	Early-pregnancy (n=1,848)	Mid-pregnancy (n=1,832)	Late-pregnancy (n=1,820)
Basic models[b]	0.32 (-0.11, 0.75)	0.51 (0.07, 0.95)*	0.30 (-0.13, 0.72)
Socio-demographic models[c]	0.29 (-0.14, 0.73)	0.41 (-0.02, 0.83)	0.24 (-0.19, 0.67)
Lifestyle models[d]	0.31 (-0.09, 0.72)	0.42 (0.001, 0.83)	0.27 (-0.13, 0.67)

Glycemic load (SDS)	Differences in systolic blood pressure in mmHg (CI 95%)		
	Early-pregnancy (n=1,848)	Mid-pregnancy (n=1,833)	Late-pregnancy (n=1,820)
Basic models[b]	0.57 (0.03, 1.12)*	0.50 (-0.05, 1.04)	0.35 (-0.17, 0.88)
Socio-demographic models[c]	0.59 (0.05, 1.13)*	0.50 (-0.04, 1.03)	0.36 (-0.16, 0.88)
Lifestyle models[d]	-0.11 (-1.14, 0.92)	-0.09 (-1.11, 0.92)	-0.02 (-1.02, 0.97)
	**Differences in diastolic blood pressure in mmHg (CI 95%)**		
	Early-pregnancy (n=1,848)	Mid-pregnancy (n=1,832)	Late-pregnancy (n=1,820)
Basic models[b]	0.24 (-0.19, 0.66)	0.16 (-0.27, 0.59)	-0.02 (-0.45, 0.40)
Socio-demographic models[c]	0.25 (-0.17, 0.67)	0.17 (-0.26, 0.60)	0.01 (-0.41, 0.42)
Lifestyle models[d]	0.96 (0.17, 1.75)*	0.87 (0.07, 1.67)*	0.53 (-0.24, 1.31)

SDS, standard deviation score. CI, Confidence Interval. [a]Values are regression coefficients (95% confidence interval) from multiple linear regression models and reflect the differences in mmHg blood pressure per one increase in standard deviation score of maternal glycemic index and glycemic load during early-pregnancy. Estimates are from multiple imputed data. [b]Basic models are adjusted for gestational age at time of intake. [c] Socio-demographic models are adjusted for maternal age, educational level, parity and gestational age at time of measurements. [d]Lifestyle models are adjusted for maternal age, educational level, parity, prepregnancy BMI, kcal, smoking habits, alcohol use, folic acid use and gestational age at time of the measurements. *P-value <0.05.

Supplementary table 8b. Sensitivity analysis: Associations of dietary glycemic index and load with uterine artery resistance index, umbilical artery pulsatility index and bilateral notching in population with study enrollment <14 weeks of gestation (n=1,867) [a].

	**Differences in UmPI (95% CI)[a]**		**Differences in UtRI (95% CI)[a]**		**Bilateral notching (95% CI)[b]**
**Glycemic index (SDS)**	Mid-pregnancy n=1,504	Late-pregnancy n=1,602	Mid-pregnancy n=1,156	Late-pregnancy n=1,222	Late-pregnancy n_{cases}=30
Basic model[c]	0.000 (-0.010, 0.009)	0.003 (-0.005, 0.011)	0.003 (-0.008, 0.003)	0.001 (-0.006, 0.003)	0.92 (0.63, 1.34)
Socio-demographic models[d]	-0.002 (-0.011, 0.008)	0.001 (-0.007, 0.009)	0.001 (-0.005, 0.004)	-0.001 (-0.005, 0.004)	0.95 (0.65, 1.40)
Lifestyle models [e]	-0.003 (-0.013, 0.006)	0.000 (-0.009, 0.008)	-0.003 (-0.008, 0.003)	-0.001 (-0.006, 0.003)	0.95 (0.78, 1.15)
**Glycemic load (SDS)**	Mid-pregnancy n=1,504	Late-pregnancy n=1,602	Mid-pregnancy n=1,156	Late-pregnancy n=1,222	Late-pregnancy n_{cases}=30
Basic model[c]	-0.003 (-0.012, 0.007)	-0.005 (-0.014 0.003)	0.002 (-0.004, 0.007)	0.001 (-0.004, 0.005)	1.02 (0.71, 1.48)
Socio-demographic models[d]	-0.002 (-0.011, 0.007)	-0.005 (-0.013, 0.003)	0.002 (-0.003, 0.008)	0.002 (-0.001, 0.004)	0.89 (0.74, 1.42)
Lifestyle models [e]	-0.008 (-0.026, 0.010)	-0.004 (-0.020, 0.012)	-0.008 (-0.019, 0.002)	-0.011 (-0.019, -0.002)*	1.08 (0.52, 2.25)

SDS, standard deviation score. CI, Confidence Interval. UmPI, umbilical artery pulsatility index. UtRI, uterine artery resistance index. [a]Values are regression coefficients (95% confidence interval) from multiple linear regression models and reflect the differences in umbilical artery pulsatility index and uterine artery resistance index per one increase in standard deviation score of maternal glycemic index and glycemic load during early-pregnancy. Estimates are from multiple imputed data. [b]Values are odds ratios (95% confidence interval) from multiple logistic regression models and reflect the difference in risks of bilateral notching per one increase in standard deviation score of maternal glycemic index and glycemic load during early-pregnancy. Estimates are from multiple imputed data. [c]Basic models are adjusted for gestational age at time of intake. [d]Socio-demographic models are adjusted for maternal age, educational level, parity and gestational age at time of measurements. [e]Lifestyle models are adjusted for maternal age, educational level, parity, prepregnancy BMI, kcal, smoking habits, alcohol use, folic acid use and gestational age at time of the measurements. *P-value <0.05.

Supplementary table 8c. Sensitivity analysis: Associations of dietary glycemic index and load with hypertensive disorder of pregnancy, gestational hypertension and preeclampsia in population with study enrollment <14 weeks of gestation (n=1,867) [a].

	**Gestational hypertensive disorders**[b]	**Gestational hypertension**[b]	**Preeclampsia**[b]
**Glycemic index (SDS)**	Odds ratio(95% CI)	Odds ratio(95% CI)	Odds ratio(95% CI)
	n_{cases}=119	n_{cases}=91	n_{cases}=28
Basic model[c]	1.01 (0.84, 1.23)	1.06 (0.85, 1.31)	0.88 (0.60, 1.30)
Socio-demographic models[d]	1.01 (0.83, 1.24)	1.07 (0.85, 1.35)	0.84 (0.56, 1.27)
Lifestyle models[c]	1.01 (0.84, 1.22)	1.06 (0.85, 1.32)	0.87 (0.60, 1.25)
**Glycemic load (SDS)**	Odds ratio(95% CI)	Odds ratio(95% CI)	Odds ratio(95% CI)
	n_{cases}=119	n_{cases}=91	n_{cases}=28
Basic model[c]	1.04 (0.86, 1.25)	1.04 (0.84, 1.28)	1.06 (0.73, 1.53)
Socio-demographic models[d]	1.05 (0.87, 1.27)	1.05 (0.85, 1.29)	1.10 (0.76, 1.59)
Lifestyle models[c]	1.04 (0.71, 1.55)	1.25 (0.80, 1.94)	0.59 (0.26, 1.33)

SDS, standard deviation score. CI, Confidence Interval. [b]Values are odds ratios (95% confidence interval) from multiple logistic regression models and reflect the difference in risks of gestational hypertensive disorders, gestational hypertension and preeclampsia per one increase in standard deviation score of maternal glycemic index and glycemic load during early-pregnancy. Estimates are from multiple imputed data. [c]Basic models are adjusted for gestational age at time of intake. [d]Socio-demographic models are adjusted for maternal age, educational level, parity and gestational age at time of measurements. [e]Lifestyle models are adjusted for maternal age, educational level, parity, prepregnancy BMI, kcal, smoking habits, alcohol use, folic acid use and gestational age at time of intake. *P-value <0.05.

Supplementary Table S9a. Sensitivity analysis: Associations of dietary glycemic index and glycemic load with systolic and diastolic blood pressure during pregnancy in complete cases[a].

**Glycemic index (SDS)**	**Differences in systolic blood pressure in mmHg (CI 95%)**		
	**Early-pregnancy**	**Mid-pregnancy**	**Late-pregnancy**
Basic models[b]	0.38 (-0.06, 0.82)<br>n=2,802	0.51 (0.11, 0.91)*<br>n=3,263	0.58 (0.19, 0.97)*<br>n=3,286
Lifestyle models[c]	-0.03 (-0.52, 0.46)<br>n=2,052	-0.13 (-0.58, 0.32)<br>n=2,357	0.10 (-0.35, 0.54)<br>n=2,394
	**Differences in diastolic blood pressure in mmHg (CI 95%)**		
	**Early-pregnancy**	**Mid-pregnancy**	**Late-pregnancy**
Basic models[b]	0.31 (-0.03, 0.65)<br>n=2,802	0.41 (0.09, 0.72)*<br>n=3,262	0.26 (-0.05, 0.58)<br>n=3,285
Lifestyle models[c]	0.15 (-0.23, 0.52)<br>n=2,052	0.25 (-0.10, 0.60)<br>n=2,356	0.25 (-0.10, 0.60)<br>n=2,393
**Glycemic load (SDS)**	**Differences in systolic blood pressure in mmHg (CI 95%)**		
	**Early-pregnancy**	**Mid-pregnancy**	**Late-pregnancy**
Basic model[b]	0.81 (0.37, 1.25)*<br>n=2,802	0.40 (-0.01, 0.80)<br>n=3,263	0.47 (0.08, 0.86)*<br>n=3,286
Lifestyle models[c]	-0.13 (-1.10, 0.84)<br>n=2,052	-0.46 (-1.34, 0.41)<br>n=2,357	-0.08 (-0.94, 0.77)<br>n=2,394
	**Differences in diastolic blood pressure in mmHg (CI 95%)**		
	**Early-pregnancy**	**Mid-pregnancy**	**Late-pregnancy**
Basic models[b]	0.35 (0.02, 0.69)*<br>n=2,802	0.06 (-0.26, 0.38)<br>n=3,262	-0.03 (-0.35, 0.28)<br>n=3,285
Lifestyle models[c]	0.76 (0.03, 1.50)*<br>n=2,052	-0.09 (-0.77, 0.59)<br>n=2,356	0.03 (-0.64, 0.69)<br>n=2,393

SDS, standard deviation score. CI, Confidence Interval. [a]Values are regression coefficients (95% confidence interval) from multiple linear regression models and reflect the differences in mmHg blood pressure per one increase in standard deviation score of maternal glycemic index and glycemic load. Estimates are from complete cases (non-imputed data). [b]Basic models are adjusted for gestational age at time of intake. [c]Lifestyle models are adjusted for maternal age, educational level, parity, prepregnancy BMI, kcal, smoking habits, alcohol use, folic acid use and gestational age at time of the measurements. *P-value <0.05.

Supplementary Table S9b. Sensitivity analysis: Associations of dietary glycemic index and glycemic load with uterine artery resistance index, umbilical artery pulsatility index and bilateral uterine artery notching in complete cases[a].

	**Differences in UmPI (95% CI)[a]**		**Differences in UtRI (95% CI)[a]**		**Bilateral uterine artery notching (95% CI)[b]**
	**Mid-pregnancy**	**Late-pregnancy**	**Mid-pregnancy**	**Late-pregnancy**	**Late-pregnancy**
**Glycemic index (SDS)**					
Basic models[c]	-0.001 (-0.008, 0.007)	0.007 (0.000, 0.013)	-0.004 (-0.008, 0.001)	-0.001 (-0.004, 0.003)	1.12 (0.84, 1.49)
	n=2,505	n=2,751	n=1,884	n=2,060	n_{cases}=48
Lifestyle models[d]	-0.005 (-0.014, 0.003)	0.003 (-0.005, 0.010)	-0.003 (-0.008, 0.002)	0.000 (-0.004, 0.004)	1.04 (0.73, 1.48)
	n=1,798	n=2,016	n=1,348	n=1,495	n_{cases}=34
**Glycemic load (SDS)**					
Basic models[c]	-0.003 (-0.010, 0.004)	0.002 (-0.004, 0.008)	0.000 (-0.004, 0.005)	0.000 (0.003, 0.004)	0.99 (0.74, 1.33)
	n=2,505	n=2,751	n=1,884	n=2,060	n_{cases}=48
Lifestyle models[d]	-0.009 (-0.026, 0.007)	0.005 (-0.009, 0.019)	0.001 (-0.009, 0.010)	-0.008 (-0.016, 0.000)	0.95 (0.85, 1.07)
	n=1,798	n=2,016	n=1,348	n=1,495	n_{cases}=34

UmPI, umbilical artery pulsatility index. UtRI, uterine artery pulsatility index. SDS, standard deviation score. CI, Confidence Interval. [a]Values are regression coefficients (95% confidence interval) from multiple linear regression models and reflect the differences in umbilical artery pulsatility index and uterine artery resistance index per one increase in standard deviation score of maternal glycemic index and glycemic load. Estimates are from complete cases (non-imputed data). [b]Values are odds ratios (95% confidence interval) from multiple logistic regression models and reflect the difference in risks of bilateral uterine artery notching per one increase in standard deviation score of maternal glycemic index and load. Estimates are from multiple imputed data. [c]Basic models are adjusted for gestational age at time of intake. [d]Lifestyle models are adjusted for maternal age, educational level, parity, prepregnancy BMI, kcal, smoking habits, alcohol use, folic acid use and gestational age at time of the measurements.*P-value <0.05.

Supplementary Table S9c. Sensitivity analysis: Associations of dietary glycemic index and glycemic load with hypertensive disorder of pregnancy, gestational hypertension and preeclampsia in complete cases[a].

	Gestational hypertensive disorders[b]	Gestational hypertension[b]	Preeclampsia[b]
	OR (95% CI)	OR (95% CI)	OR (95% CI)
**Glycemic index (SDS)**			
Basic models[c]	1.00 (0.87, 1.14)	1.02 (0.87, 1.19)	0.92 (0.71, 1.20)
	n_{cases}=224	$n_{cases=}$166	$n_{cases=}$58
Lifestyle models[d]	0.99 (0.94, 1.05)	0.97 (0.91, 1.04)	0.87 (0.63, 1.19)
	n_{cases}=154	n_{cases}=116	n_{cases}=42
**Glycemic load (SDS)**			
Basic model[c]	1.04 (0.91, 1.19)	1.03 (0.88, 1.20)	1.06 (0.83, 1.37)
	n_{cases}=224	n_{cases}=166	n_{cases}=58
Lifestyle models[d]	0.98 (0.69, 1.38)	1.06 (0.72, 1.57)	0.78 (0.42, 1.46)
	n_{cases}=514	n_{cases}=116	n_{cases}=42

SDS, standard deviation score; CI, Confidence Interval. [b]Values are odds ratios (95% confidence interval) from multiple logistic regression models and reflect the difference in risks of gestational hypertensive disorders, gestational hypertension and preeclampsia per one increase in standard deviation score of maternal glycemic index and glycemic load. Estimates are from complete cases (non-imputed data). [c]Basic models are adjusted for gestational age at time of intake. [d]Lifestyle models are adjusted for maternal age, educational level, parity, prepregnancy BMI, kcal, smoking habits, alcohol use, folic acid use and gestational age at time of intake. *P-value <0.05.
